# Microglial P2X4 receptors promote ApoE degradation and contribute to memory deficits in Alzheimer’s disease

**DOI:** 10.1007/s00018-023-04784-x

**Published:** 2023-05-05

**Authors:** Jennifer Hua, Elvira Garcia de Paco, Nathalie Linck, Tangui Maurice, Catherine Desrumaux, Bénédicte Manoury, François Rassendren, Lauriane Ulmann

**Affiliations:** 1 IGF, Univ Montpellier, CNRS, INSERM, Montpellier, France; 2 LabEx Ion Channel Science and Therapeutics, Montpellier, France; 3 MMDN, Univ Montpellier, EPHE, INSERM, Montpellier, France; 4 Institut Necker Enfants Malades, INSERM, CNRS, Université de Paris, Paris, France

**Keywords:** Purinergic signaling, Lysosome, Cathepsin, Proteomic, Soluble Aß peptide

## Abstract

Numerous evidences support that microglia contributes to the progression of Alzheimer’s disease. P2X4 receptors are ATP-gated channels with high calcium permeability, which are de novo expressed in a subset of reactive microglia associated with various pathological contexts, contributing to microglial functions. P2X4 receptors are mainly localized in lysosomes and trafficking to the plasma membrane is tightly regulated. Here, we investigated the role of P2X4 in the context of Alzheimer’s disease (AD). Using proteomics, we identified Apolipoprotein E (ApoE) as a specific P2X4 interacting protein. We found that P2X4 regulates lysosomal cathepsin B (CatB) activity promoting ApoE degradation; *P2rX4* deletion results in higher amounts of intracellular and secreted ApoE in both bone-marrow-derived macrophage (BMDM) and microglia from APP^swe^/PSEN1^dE9^ brain. In both human AD brain and APP/PS1 mice, P2X4 and ApoE are almost exclusively expressed in plaque-associated microglia. In 12-month-old APP/PS1 mice, genetic deletion of *P2rX4* reverses topographical and spatial memory impairment and reduces amount of soluble small aggregates of Aß1-42 peptide, while no obvious alteration of plaque-associated microglia characteristics is observed. Our results support that microglial P2X4 promotes lysosomal ApoE degradation, indirectly altering Aß peptide clearance, which in turn might promotes synaptic dysfunctions and cognitive deficits. Our findings uncover a specific interplay between purinergic signaling, microglial ApoE, soluble Aß (sAß) species and cognitive deficits associated with AD.

**Supplementary Information:**

The online version contains supplementary material available at 10.1007/s00018-023-04784-x.

## Introduction

Alzheimer’s disease (AD), a slowly progressive, irreversible and incurable neurodegenerative disease, is the most common form of dementia in human. The main pathological hallmarks of AD are amyloid-ß (Aß) accumulation in plaques, hyperphosphorylated Tau aggregation in neurofibrillary tangles, neuronal loss, brain atrophy and gliosis [1]. For decades, AD was mainly considered as a neuronal disease, glial cells being only considered as reacting to neuronal alterations. This neurocentric view considerably evolved in the past 10 years, with both genetic and functional studies showing that neuroinflammation contributes significantly to the onset and progression of AD [2]. Indeed, genome-wide association studies (GWAS) support that approximately 50% of the susceptibility genes associated with AD are related to glial and vascular cells and point towards innate immune system involvement [3–5]. In the central nervous system (CNS), inflammation is mainly driven by two cell types, microglial cells and astrocytes. Microglia, the brain resident macrophages, are the main immunocompetent cells, which in the healthy brain, have different homeostatic functions such as monitoring neuronal activity, shaping dendritic spines, and even influencing synaptic activity [6]. In pathological conditions, microglia enter into reactive states characterized by a transcriptional and functional remodeling. Using single cell RNAseq analysis, recent studies revealed that microglial reactivity evolves along the disease progression, generating microglial diversity and culminating with the so-called disease-associated microglia (DAM) signature characterized, among others, by the upregulation of many genes identified by GWAS such as *ApoE* and triggering receptor expressed on myeloid cells 2 (*Trem2*) [7, 8].

Three *ApoE* alleles exist in the human population, ε2, ε3 and ε4, with ε4 being the strongest genetic risk factor of sporadic AD identified so far [9]. One of the proposed mechanisms by which ApoE could favor AD is through a direct interaction between Aß and ApoE. Studies have shown that ApoE can impact both Aß seeding, fibrillogenesis and clearance in an isoform-dependent manner [10, 11], ApoE4 being more prone to facilitate seeding and to reduce Aß clearance [12, 13]. ApoE functions in the CNS are nonetheless diverse and complex and likely contribute to AD through additional mechanisms. Recent data particularly revealed that both Trem2 and ApoE are critical regulators of microglial switch from homeostatic to neurodegenerative phenotype [14, 15] and genetic ablation of either gene results in a larger proportion of microglia in homeostatic state in mouse models of AD [15, 16].

Purinergic signaling is central to microglial biology in both healthy and pathological conditions [17]. Indeed, microglia express a large repertoire of purinergic receptors as well as different proteins involved in ATP release or degradation [17]. Microglial purinergic receptor expression is highly dependent on the state of microglia. In homeostatic state, *P2ry12* gene is among the most expressed, while its expression is strongly down regulated in reactive states [18]. Conversely, P2X4 receptor, an ATP-gated channel, is not present in homeostatic microglia but its expression is induced upon activation [19].

In reactive microglia, P2X4 receptors have been linked to different functions and pathologies. In neuropathic pain models, de novo P2X4 expression in spinal cord microglia enhances local network excitability and promotes pain [20]. Similarly, following a *status epilepticus*, P2X4 hippocampal microglia likely contribute to microglial-evoked neuroinflammation and neuronal death [21]. Generally, pharmacological or genetic blockade of P2X4 receptors has beneficial effects in different acute CNS pathologies [22]. P2X4 have a high calcium permeability and their trafficking to the plasma membrane is tightly regulated, most of the protein being localized in lysosomes. Mice expressing internalization-deficient P2X4 receptor show that in an ALS (Amyotrophic lateral sclerosis) mice model, P2X4 is instrumental for motor symptoms, disease progression and survival [23]. Yet, whether P2X4 have detrimental effects in other slowly progressing neurodegenerative disease remains to be elucidated. Here, we show that in myeloid cells, P2X4 specifically interact with ApoE and triggers its degradation by regulating CatB activity. In APP/PS1 mice, a murine model AD, P2X4 is almost exclusively expressed in plaque-associated microglia. Genetic deletion of *P2rX4* in APP/PS1 results in higher amount of intracellular and secreted ApoE in both BMDM and microglia, in a lower amount of soluble forms of Aß1–4 and reverses spatial learning deficit associated with APP/PS1 expression. We also provide evidence that in human AD brain, P2X4 and ApoE are co-localized in microglia associated with amyloid plaques. Our results support that in AD microglial P2X4 promotes lysosomal ApoE degradation, indirectly altering Aß peptide clearance, which in turn might promote synaptic dysfunctions and memory impairment.

## Methods

### Animals

Mice carrying a targeted null mutation of the *P2rX4* gene were described elsewhere [24]. Briefly, a *E. Coli* ß-galactosidase (LacZ)-neomycin cassette was inserted in place of the first coding exon of the *P2RX4* gene. In the resulting allele, the *P2rX4* promoter drives ß-galactosidase expression. Chimeric mice were generated and crossed with C57BL/6 females to generate heterozygotes, which were then intercrossed to give rise to overtly healthy offspring in the expected Mendelian ratio. In the present study, mice were backcrossed for at least 20 generations and then maintained as separate *P2rX4* knockout (P2X4 KO) and wild-type (WT) lines. As described previously [25, 26], P2X4 KO mice carry a P2X7 passenger mutation originating from the 129 genetic background of ES cells. This implies that in all experiments comparing P2X4 KO to either C57/Bl6 or APP/PS1, P2X7 harbored either 451L or 451P residue, respectively. All experiments followed European Union (Council directive 86/609EEC) and institutional guidelines for laboratory animal care and use. Institutional license for hosting animals was approved by the French Ministry of Agriculture (No. D34-172-13). The Tg(APPswe,PSEN1dE9)85Dbo mice [27] (APP/PS1) were obtained from the Jackson Laboratory (JAX stock #034,832) and bred as heterozygotes to C56 Bl6/J or P2X4 KO mice. All experiments using APP/PS1 and APP/PS1xP2X4 KO mice were carried out at 12 months of age.

### Behavioral experiments

*Hamlet test* The Hamlet test was performed as previously described [28, 29]. Briefly, the device consisted of a 1.6 m diameter apparatus with an agora in the center and five corridors expanding toward different compartments, called houses. Each house has a different interest: drink, eat, run, hide or interact with a stranger mouse. Mice were trained in group and were allowed to go freely in the apparatus for 4 h per day during 12 days. Probe tests were performed 72 h and 96 h after the last training day, in water-deprived or non-water deprived conditions, respectively. For water-deprived condition, water bottles were removed from mice housing cages 15 h before the test. Mice were placed in the agora for 10 min and exploratory behaviors were video-tracked and analyzed with the Viewpoint software as latency time and number of errors to go to the drink house.

*Morris water maze* The Morris water maze test was performed in a 1.4 m diameter (40 cm height) circular tank with extra maze cues. Tank was filled with 22 °C water containing non-toxic lime carbonate to make it opaque. A 10 cm-diameter circular platform was immerged under water, thus not visible to mice. Mice were trained three times a day for six consecutive days. They were allowed a free 90 s swim in order to find the platform. If by that time, mice did not find the platform, they were gently place on it and allowed to stay there for 20 s. Probe test were performed 48 h after the last training day. The platform was removed and mice swam for 60 s. A video camera recorded the probe test and analysis was performed using the Viewpoint software.

*Locomotor activity* Mice were place in a square open field box for 10 min. Viewpoint software tracked animals and calculated the distance traveled.

#### Tissue preparation

Mice were euthanized with Euthasol (300 mg/kg) and perfused with PBS. Brains were either collected and fixed in 4% paraformaldehyde (PFA) at 4 °C overnight or stored at – 80 °C.

#### Cell culture and transfection

COS-7 cells were cultured in Dulbecco’s Modified Eagle Medium (DMEM) containing 2 mM glutaMAX supplemented with 10% fetal bovine serum (FBS) and 100 U/mL penicillin, 100 µg/mL streptomycin, and kept at 37 °C with 5% CO_2_. Before transfection, cells were plated at 70% confluence in 6-well plates. Transfection was carried out using Lipofectamine 2000 (Thermo Fisher Scientific), with the following DNA amount: 150 ng *ApoE*, 100 ng *P2rX4*, 100 ng *P2rX4-K69A* and 100 ng *P2rX2.* All plasmids used in this study where either provided by Dr G. Buell (P2X2, P2X7 451L), by Pr. S Adriouch (P2X7 451P) or cloned by RT-PCR in appropriate expression vector (mouse P2X4, mouse ApoE). Medium was changed for Hank’s Balanced Buffered Solution (HBSS) (Gibco) 48 h after transfection and supernatants and cell extracts were collected the next day as described below.

#### BMDM culture

BMDM were obtained from mice femur and tibia bone marrow and cultured in 30% L929 cell conditioned medium produced in the lab and 70% DMEM containing 2 mM glutaMAX (Thermo Fisher Scientific), supplemented with 10% fetal bovine serum (FBS, Biowest) and 100 U/mL penicillin 100 µg/mL streptomycin. Cells were mechanically dissociated and plated and medium was changed every 3 days. BMDM from CatB-deficient mice [30] were kindly provided by Dr. Bénédicte Manoury (Hôpital Necker Enfants Malades, Paris).

#### Pharmacological treatment

Macrophages were plated in a 12-well plate at 10^6^ cells per well and treated with 10 µM E64 (Tocris, 5208), 10 µM calpain inhibitor III (Cayman Chemicals, 14,283) or 20 µM Z-Phe-Ala-FMK CatB inhibitor (Santa-Cruz, sc3131) in HBSS overnight.

#### Primary and secondary antibodies

*Immunocytochemistry* goat anti-ApoE (1:1000, Millipore, AB947), goat anti-Cathepsin B (1:1000, R&D systems, AF965), rabbit anti-P2X4 (1:200, Sigma, HPA039494), donkey anti-CD68 (1:300, Biorad, MCA1957A488T), mouse anti-6E10 (1:500, Biolegend, SIG-39320–0200), rabbit anti-Iba1 (Ionized calcium-binding adapter molecule 1, 1:2000, Wako, MNK4428), rat anti-P2X4 (1:200, kindly provided by Dr. Nolte [31]), donkey anti-rat-A594 (1:500, Jackson Immunoresearch, 712-586-150), donkey anti-goat-A488 (1:2000, Molecular probes, A11055), donkey anti-rabbit-A557 (1:2000, R&D systems, NL004), goat anti-rabbit-A488 (1:2000, Molecular probes, A11034).

*Western blot* mouse anti-tubulin (1:5000, Sigma, T9026), rabbit anti-HA (1:500, Invitrogen, 715,500), rabbit anti-P2X4 (1:500, Alomone Labs, APR-002), anti-HA beads (1:500 Santa-Cruz Biotechnology), horse anti-mouse-HRP (1:2000, Cell signaling, 7076S), goat anti-rabbit-HRP (1:2000, Jackson Immunoresearch, 111-035-144), donkey anti-goat-HRP (1:2000, Jackson Immunoresearch, 705–035-003).

#### Western blot

For BMDM culture cells, supernatants were collected in Amicon column (Millipore, UFC5010BK) and centrifuged at 14,000g for 30 min at 4 °C. Column fractions were then collected and constituted the supernatant fractions of our cells. Cells were homogenized in lysis buffer (100 mM NaCl, 20 mM HEPES, 5 mM EDTA, 1% IGEPAL containing protease inhibitors). For cortex samples, dissected cortices were mechanically homogenized in 1% Triton lysis buffer (100 mM NaCl, 20 mM HEPES, 5 mM EDTA, 1% Triton X-100 containing protease inhibitors) before homogenization on a wheel at 4 °C for 1 h. Protein extracts were then centrifuged at 15,000g at 4 °C for 10 min. After measuring protein concentration using Bradford technique, LDS sample buffer and 10% β-mercapto-ethanol were added. Proteins were then separated by reducing 4–12%, SDS-PAGE and transferred to a nitrocellulose membrane. The membrane was blocked with PBS with 0.1% Tween 20 (PBST) containing 5% non-fat dry milk overnight at 4 °C. The membrane was then incubated overnight at 4 °C with the indicated antibodies. After three washes in PBST, the membrane was then treated for 45 min at room temperature with the appropriated HRP-conjugated secondary antibody: Proteins were visualized using an ECL + detection kit (Amersham) and imaged using a Chemidoc Touch Imaging system (Biorad). Densitometry was analyzed using the ImageLab software.

Western blot data were quantified by expressing the densitometric ratio of the protein of interest over that of tubulin. In the case of ApoE, because of high inter-experiment variability, densitometric ratio of ApoE were normalized to an arbitrary value of 1 assigned to WT or untreated conditions. A comparison of unnormalized versus normalized data is shown in Sup Fig. 3.

#### Human tissue

Frozen brain samples from human tissue were obtained by the IHU-A-ICM-Neuro-CEB brain bank (Hôpital de la Pitié-Salpétrière, Paris). For immunostaining, cortex slice arrived frozen and mounted on microscope slides.

#### Aβ ELISA (Enzyme-Linked Immuno Sorbent Assay)

Cortices were homogenized in Tris buffer (Tris 1 M, pH 7.6-SDS 2%) containing protease and phosphatase inhibitors and 1 mM AEBSF. Homogenates were then sonicated at 40 mV for 10 s and centrifuged at 13,000g for 30 min at 4 °C. Supernatants were then collected and constituted the soluble fraction of the sample. Quantification of soluble Aβ peptide was performed using an ELISA kit (Thermofisher, KHB3441).

#### Immunostaining

Tissues were cut using a vibratome into 40 µm sections, rinsed with PBS and blocked with 10% goat serum diluted in a 0.1% Triton X-100 solution for 2 h. Appropriate primary antibody was added overnight at 4 °C. After rinsing, slices were incubated for 2 h with corresponding secondary antibody. Antibodies were diluted in PBS with 0.1% Triton X-100. Amylo Glo (Biosensis, TR300-AG) was used for amyloid plaque staining according to the provided instructions. Briefly, before immunostaining, brain sections were transferred in a 70% ethanol solution, rinsed with distilled water and incubated with Amylo Glo for 10 min. Before mounting, sections were incubated with 1 × True Black^®^ (Biotium) to quench lipofusin autofluorescence. After rinsing, sections were mounted with Fluorescent Mounting medium (Dako) and observed on an AxioImager Z1 apotome (Zeiss).

BMDM cells were fixed in 1% PFA, washed and incubated with 10% goat or donkey serum in PBS containing 0.1% Triton X-100 for 30 min. Cells were then incubated with primary antibodies for 2 h, washed and incubated with secondary antibodies for 1 h before mounting.

Human tissues were fixed with 4% PFA and incubated with 10% goat or donkey serum in PBS containing 0.1% Triton X-100. Primary antibodies were directly put on slides in PBS containing 0.1% Triton X-100 overnight. After washing three times in PBS, tissues were incubated with the secondary antibodies for 2 h.

#### Amyloid plaque quantification

Amyloid plaques size was quantified using Thioflavine T staining. Brain section was stained with 100 µg/mL Thioflavine T (Sigma T3516) for 15 min, rinsed with ethanol 70% for 5 min once and with PBS three times. Brain sections were mounted and images were acquired using a Zeiss AxioImager Z1 microscope. Plaque size was quantified using the threshold function in ImageJ. Then, frequency was calculated using the frequency function in Excel. For each animal, 5 brains sections were analyzed.

#### Microglia area quantification

Brain sections were stained with AmyloGlo^®^ and ionized calcium-binding adapter molecule 1 (Iba1) antibody in order to stained amyloid plaques and microglia. For quantification, fields containing plaques were randomly chosen; six fields per section, five sections per animals were acquired using a Zeiss AxioImager Z1microscope. For each amyloid plaque, the field of interest analyzed is defined by a perimeter that is proportional to the plaque size: the perimeter is calculated with a radius equal to four time the radius of the amyloid plaque. The Iba1 area is quantified in the zone using the threshold function in ImageJ.

#### Cathepsin B fluorescent activity

BMDM were plated in a 96-well plate at 10^5^ cells per well and incubated with 100 µM of the CatB substrate Z-RR-AMC (Enzo Life Sciences, BML-P137) for 1 h and 2 h before reading fluorescence (ex 365, em 440) on a plate reader (Flexstation 3, Molecular Devices). For CatB activity assessment by microscopy, macrophages were plated in a 24-well plate containing cover slips and incubated with the Magic Red CatB substrate (1:250, Bio-Rad, ICT938) for 2 h. Cells were then fixed and mean fluorescence intensity in cells was quantified using the ImageJ software.

#### Membrane solubilization

Plasma membrane-enriched protein fractions were prepared from freshly isolated mouse BMDM. BMDM were detached by cell scraping, counted, pelleted and homogenized in a solubilization buffer (0.32 M sucrose, 10 mM HEPES, 2 mM EDTA and complete protease inhibitor cocktail, pH 7.4) with 150 strokes of a Potter–Elvehjem homogenizer (Dominique Dutcher). The homogenates were centrifuged 20 min, at 1000 g at 4 °C to eliminate the debris and the supernatant was centrifuged 1 h at 70,000g at 4 °C. The supernatant was discarded and the resulting pellet was solubilized in a set of detergent buffers of variable stringency (Complexiolyte (Logopharm)), CL48 was chosen for further experiments due to its physiological stringency and after analysis of its solubilization efficiency) supplemented with protease inhibitors (Roche) for 2 h at 4 °C. Insolubilized material (pellet; particles > 336 S) was removed by centrifugation (30,000g, 18 min, 4 °C) leaving micelles with an estimated size (diameter) of up to 75 nm in the supernatant.

#### Purification of P2X4 receptor complex by immunoprecipitation and analysis by mass spectrometry

Freshly prepared solubilized proteins were incubated o/n at 4 °C with affinity-purified rabbit anti-P2X4 antibody (Alomone Labs) crosslinked to magnetic beads (Dynabeads, Invitrogen). The flow through was discarded and the beads washed 5 times with wash buffer (CL48 diluted ¼ in PBS and supplemented with complete protease inhibitor cocktail (Roche)) and sample buffer (Invitrogen) was added to separate the protein complexes from the beads. Eluates were shortly run on SDS/PAGE gels, Coomassie blue stained and sliced according to molecular mass. Further treatments and tandem mass spectrometry were performed at the Harvard Medical School Taplin Biological Mass spectrometry facility, Boston, MA, USA.

Freshly prepared solubilized proteins were incubated o/n at 4 °C with affinity-purified rabbit anti-P2X4 antibodies (1:500, Alomone Labs, APR-002) crosslinked to magnetic beads (Dynabeads, Invitrogen). The flowthrough was discarded and the beads washed 5 times with wash buffer (CL48 diluted 1:4 in PBS and supplemented with complete protease inhibitor cocktail (Roche)) and sample buffer (Invitrogen) was added to separate the protein complexes from the beads. Eluates were shortly run on SDS/PAGE gels and blue stained before the digestion with trypsin. The extracted peptides were dissolved and loaded onto a precolumn of an UltiMate 3000 HPLC system (Dionex). Eluates were electrosprayed into a mass spectrometer (LTQ-Orbitrap XL, Thermo Fisher Scientific). Peptides were separated onto a capillar column (phase inverse C18, Pepmap^®^, Dionex) in a gradient of 0–40% of B in 60 min (*A* = 0.1% formic acid, 2% acetonitrile; *B* = 0.1% formic acid in acetonitrile) in a flow of 300 nl/min. Spectra were recorded with the Xcalibur 2.0.7 software (Thermo Fisher Scientific). Data were analyzed with the Proteome Discoverer v1.4 software (Thermo Fisher Scientific) and Mascot search engine (Matrix Science) v2.4. Extracted MS/MS spectra were searched against the CPS_mouse 2013_05 database.

#### Co-immunoprecipitation

Experiments with BMDM were carried out with membrane-enriched protein fractions (see protocol above). COS-7 cells were homogenized in lysis buffer (100 mM NaCl, 20 mM HEPES, 5 mM EDTA, 1% NP-40 and complete protease inhibitor cocktail pH 7.4) 48 h after transfection. Lysates were clarified by centrifugation. Protein concentration of the lysates was determined using a protein assay kit (Bio-Rad) and were incubated on a rotating wheel with specific antibodies crosslinked to magnetic beads (Dynabeads, Invitrogen) for 4 °C, o/n. After five washes in lysis buffer, bound proteins were eluted with sample buffer (Invitrogen).

#### Cytometry

Mice were perfused with PBS and cortices were collected and dissociated using the Neural Tissue Dissociation Kit P (Miltenyi, 130-092-628) combined with the gentleMACS Octo Dissociator with heaters, as indicated by the supplier. After dissociation, myelin was removed using the Debris Removal Solution (Miltenyi, 130-109-398). Cells were then incubated with Fc bloc (1:100, BD Pharmingen, 553,142) for 10 min on ice and stained with CD11b-PE (1:100, BD Pharmingen, 557,397) for 30 min on ice. Cells were first discriminated by size and granularity. Microglia were then sorted using a laser with a 561 nm excitation wavelength and a 582 nm filter, with a purity ≥ 95% (Sup. Fig. 1). Afterward, sorted microglia were homogenized as described above and used in Western blot experiments.

#### Quantitative PCR analysis

Total RNA from macrophage cultures was extracted with RNeasy Mini Kit (Qiagen). A measure of 2 µg of total RNA were reverse transcribed using random hexamers and SuperScript III First-Strand synthesis System (Invitrogen) according to the manufacturer’s instructions. Realtime PCR was performed by using SYBR Green dye detection according to the manufacturer’s instruction (SYBR Green PCR Master Mix, Roche) on a LightCycler480 system. PCR reactions were performed in a 10 ml volume containing 2.5 ml of diluted RT product, 1 ml of forward and reverse primers and 5 ml of PCR master mix. Negative controls using non-reverse-transcribed RNA were performed simultaneously. For each reaction, Cq was determined using the 2nd Derivative Max tool of LightCycler480 software. The relative ratios of specifically amplified cDNAs were calculated using the DCq method [32]. RNAs from three independent cultures were used. All experimental conditions were processed at the same time.

#### Measurement of lysosomal pH

BMDM were plated on 96-well plates and incubated with Lysosensor^™^ (3 µM, Invitrogen, L7545) for 3 min at 37 °C. Cells were then rinsed with PBS twice. A calibration curve of the intensity fluorescence as a function of pH was made. In order to do so, after incubation with Lysosensor^™^, cells were incubated with determined pH solution for 10 min at 37 °C. Fluorescence was determined using a plate reader Spark (Tecan) using 340 nm and 380 nm excitation wavelength. Then, ratio between fluorescence intensity resulting from the 340 nm and 380 nm excitation were calculated and pH was determined using the calibration curve.

#### Statistics

Statistics tests were performed using the GraphPad Prism9 software. After checking that all parametric assumptions were met, data were analyzed using Student's or ANOVA test. When the assumptions were not met, Wilcoxon signed-rank or Kruskal–Wallis test were used. For pharmacological treatment, data were paired. For each graph, mean ± SEM are indicated.

## Results

### P2X4 interacts with ApoE in myeloid cells

**Fig. 1:**
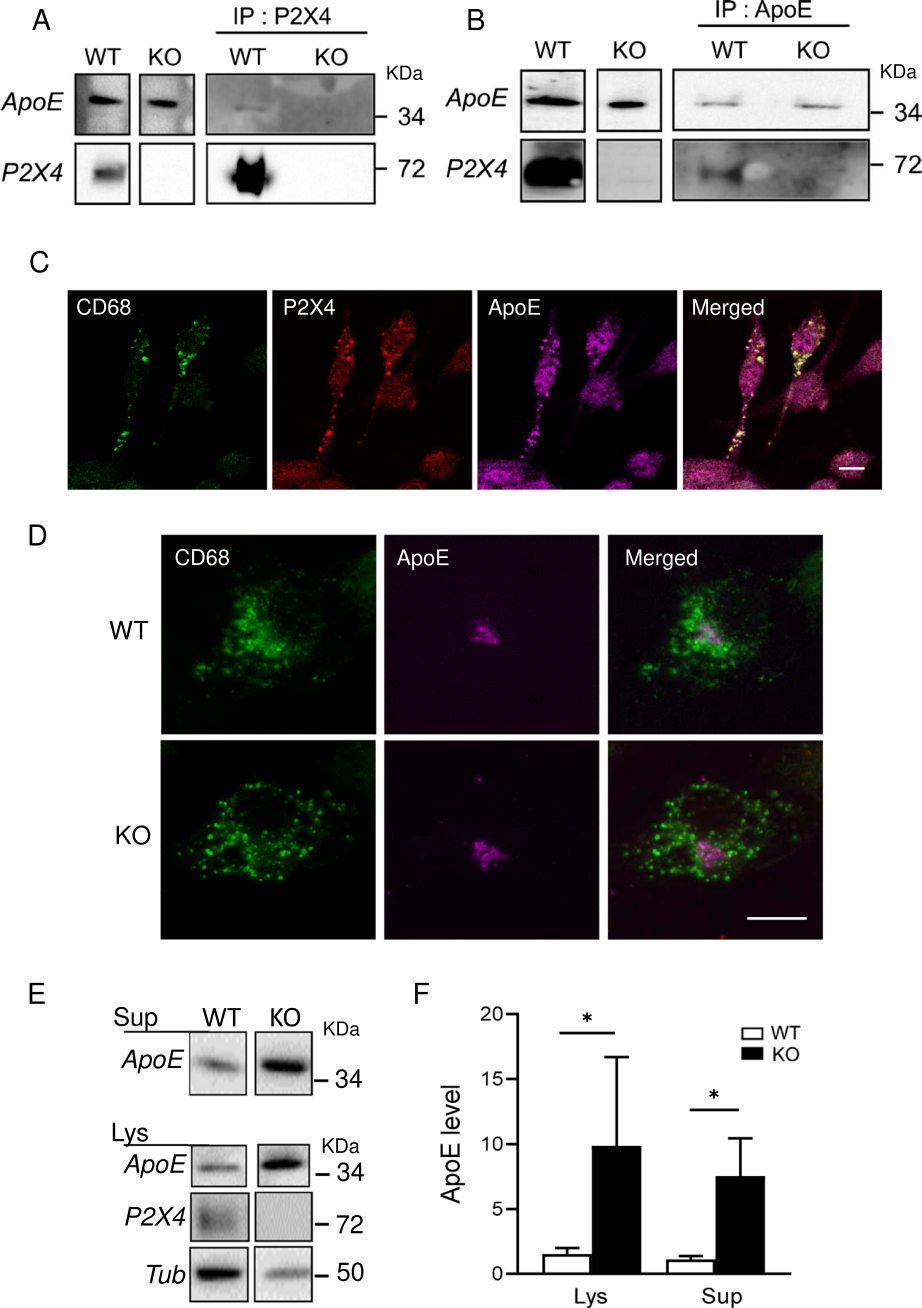
P2X4 interacts with ApoE in BMDM endo-lysosomal compartments and reduces its amount compared to P2X4-deficient cells. **A**, **B** Co-immunoprecipitation of P2X4 and ApoE. BMDM membrane extracts from WT and KO mice were immunoprecipitated (IP) with anti-P2X4 (**A**), or ApoE antibodies (**B**). Immunoprecipitated proteins were separated by electrophoresis and immunoblotted with either anti-ApoE (top row) or anti-P2X4 (bottom row) antibodies. **C** Representative immunofluorescence image showing the co-localization of the lysosomal marker CD68 (green), P2X4 (red) and ApoE (purple) in BMDM cells. Scale bar 5 µm. **D** Representative immunofluorescence image showing a similar localization of ApoE in CD68 + lysosomes in WT and P2X4KO BMDM cells. Scale bar 5 µm. All immunocytochemistry experiments were replicated at least three times. **E** Representative Western blot of ApoE in BMDM culture supernatants (Sup) or cell lysates (Lys) from WT and KO mice. **F** Quantitative Western blot analysis presented in E. A significant increase of ApoE is observed in both KO cultures supernatants and in cell lysates. *N* = 6 independent experiments, *p < 0.05, unpaired *t* test

P2X4 receptors present a complex trafficking regulation with a prominent intracellular localization in the endo-lysosomal compartment [33] and different studies suggested that P2X4 receptor can regulate specific functions such as lysosomal secretion or calcium flux. To identify potential P2X4 partners involved in endo-lysosomal functions, we developed an approach based on intracellular membrane compartment enrichment, antibody-based affinity purification of native P2X4 receptors, and mass spectrometry (see “Methods”). Affinity purification was performed on bone-marrow-derived macrophages (BMDM) from both WT and P2X4 KO mice. Among the different proteins interacting specifically with P2X4, ApoE was the only one with significant coverage found across two independent experiments (Sup. Fig. 2). In BMDM, P2X4-ApoE interaction was further confirmed by immunoprecipitation using either P2X4 or ApoE antibodies (Fig. 1A, B).

**Fig. 2:**
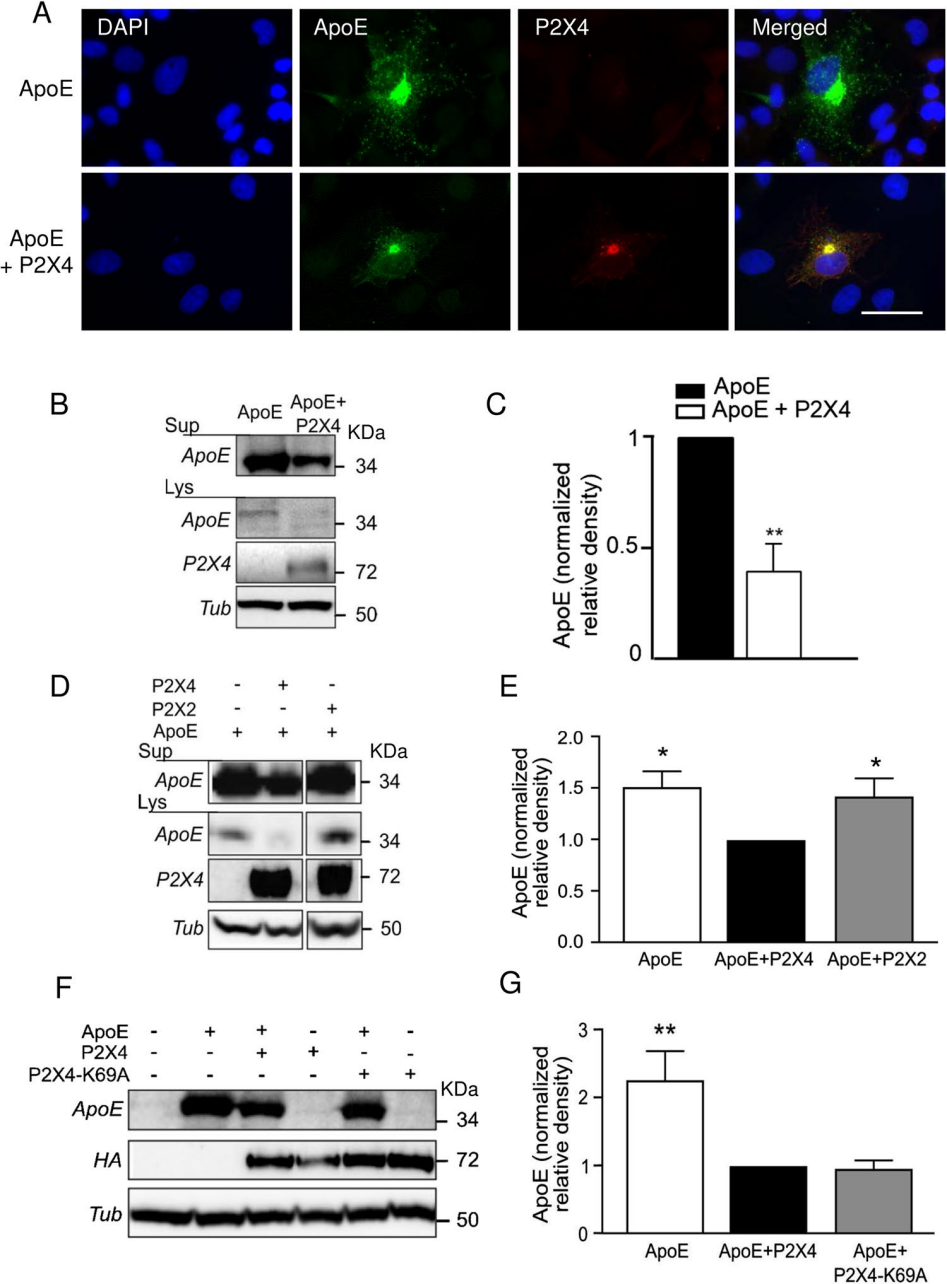
Interaction between P2X4 and ApoE is recapitulated in recombinant system. **A** Representative immunofluorescence of ApoE (green), P2X4 (red), and DAPI (blue) in ApoE or ApoE + P2X4 co-transfected COS-7 cells. Both ApoE and P2X4 co-localize in intracellular compartments. Images are representative of *N* ≥ 3 independent experiments. Scale bar 10 µm. **B, C** Comparison of ApoE expression upon co-transfection with P2X4. COS-7 cells were transfected with ApoE alone or in combination with P2X4. **B** Expression of ApoE was analyzed by Western blot in both cell culture supernatants and cell lysates. **C** Quantitative analysis shows that in the presence of P2X4, amounts of ApoE is reduced in both culture supernatant (Sup) and cell lysates (Lys). *N* = 3 independent experiments, **p < 0.01, One sample *t* test compared to theoretical value of 1. **D, E** Comparison of ApoE expression upon co-transfection with P2X4 or P2X2. **D** Expression of ApoE was analyzed by Western blot in both cell culture supernatants and cell lysates. **E** Quantitative analysis of ApoE in supernatant shows that co-expression with P2X4 reduces the expression of ApoE, whereas that of P2X2 has no effect. *N* = 6 independent experiments, *p < 0.05, One sample Wilcoxon compared to theoretical value of 1 attributed to ApoE + P2X4. **F, G** P2X4 activity is not necessary to reduce ApoE levels. **F** Expression of ApoE was analyzed by Western blot in cell culture supernatants of cells transfected with ApoE alone or in combination of either P2X4 or P2X4-K69A, an ATP-binding site dead mutant. **G** Quantitative analysis shows that both and P2X4 P2X4-K69A significantly reduces the ApoE levels to the same extend. *N* = 8 independent experiments, **p < 0.01, one sample *t*-test compared to theoretical value of 1 attributed to ApoE + P2X4

Immunocytochemistry revealed that in BMDM, P2X4 and ApoE co-localized in the endo-lysosomal compartment, as both proteins also co-localized with CD68, a specific endo-lysosomal marker (Fig. 1C). This is also consistent with the known localization of both proteins in lysosomes [33, 34].Yet, both ApoE and P2X4 have their respective, non-overlapping intracellular distribution. In addition, we did not observe apparent alteration of the intracellular localization of ApoE between WT and P2X4 KO BMDM **(**Fig. 1D**)** suggesting that P2X4 and ApoE do not co-traffic in the endo-lysosomal compartment. In lysosomes, P2X4 is protected from degradation by the complex glycosylation of its intra-lumen loop [33], contrary to ApoE which undergo lysosomal degradation [35]. We thus ask whether P2X4 could protect ApoE from lysosomal degradation. To that aim, we compared the expression of ApoE in BMDM from WT and P2X4 KO mice by Western blotting. Since ApoE is secreted by BMDM, amounts of ApoE were determined in both cell lysates and cell culture supernatants. As shown in Fig. 1E, F, BMDM from KO mice express much higher levels of ApoE in both cell lysates and supernatants. Levels of ApoE in WT BMDM were 1.53 ± 0.46 and 1.1 ± 0.27 in lysate and supernatant, respectively. For P2X4 KO BMDM levels of ApoE were 9.87 ± 6.81 and 7.54 ± 2.91 in lysate and supernatant, respectively. By comparison normalized data show the same results but with lower variability (Sup. Fig. 3). Identical results were obtained in the lysate of primary culture of microglia from WT and KO mice (Sup. Fig. 4). RT-qPCR transcriptional analysis of ApoE mRNA from WT and KO BMDM did not reveal any difference (Sup. Fig. 5) further supporting that the physical interaction between P2X4 and ApoE modulates ApoE degradation. These effects of P2X4 on ApoE were reproduced in transfected COS-7 cells. Co-transfected cells show clear intracellular co-localization of P2X4 and ApoE (Fig. 2A). In addition, no striking difference in the intracellular distribution of ApoE could be observed in the presence or absence of P2X4. As observed in BMDM, P2X4/ApoE co-transfected COS-7 cells presented significant lower amounts of ApoE in both lysates and supernatants compared to cells transfected with ApoE alone (Fig. 2B, C).

We next examined whether the effect of P2X4 subunit on ApoE was specific. To address this question, ApoE contents were analyzed as above in COS-7 cells expressing ApoE alone or co-transfected with either P2X4 or P2X2. As shown in Fig. 2D, E, as expected, co-expression of ApoE and P2X4 induced a reduction of both cellular and secreted ApoE as compared to cells expressing ApoE alone (0.95 ± 0.3 vs 1.34 ± 0.3), while co-expression of ApoE and P2X2 did not alter ApoE levels (1.18 ± 0.24 vs 1.34 ± 0.3, unnormalized values). This indicates that ApoE regulation was specific to P2X4 subunit.

Finally, we asked whether P2X4-mediated ApoE downregulation was dependent on its channel activity. As above, levels of ApoE were measured in lysates and supernatants from COS-7 cells expressing ApoE alone or co-transfected with either P2X4 or P2X4-K69A, a mutant form of the receptor unable to bind ATP. As shown in Fig. 2F, G, levels of ApoE were the same in cells transfected with P2X4 or P2X4-K69A, but significantly lower than in cells expressing ApoE alone. These results suggest that P2X4 receptor activity is not necessary to drive downregulation of ApoE, although we cannot exclude that, in lysosome, other intracellular signaling molecules such as pH or phosphatidylinositol 4,5-biphosphate (PI(4,5)P_2_) could directly gate P2X4 channel.

### P2X4 induces cathepsin B-dependent ApoE degradation

**Fig. 3:**
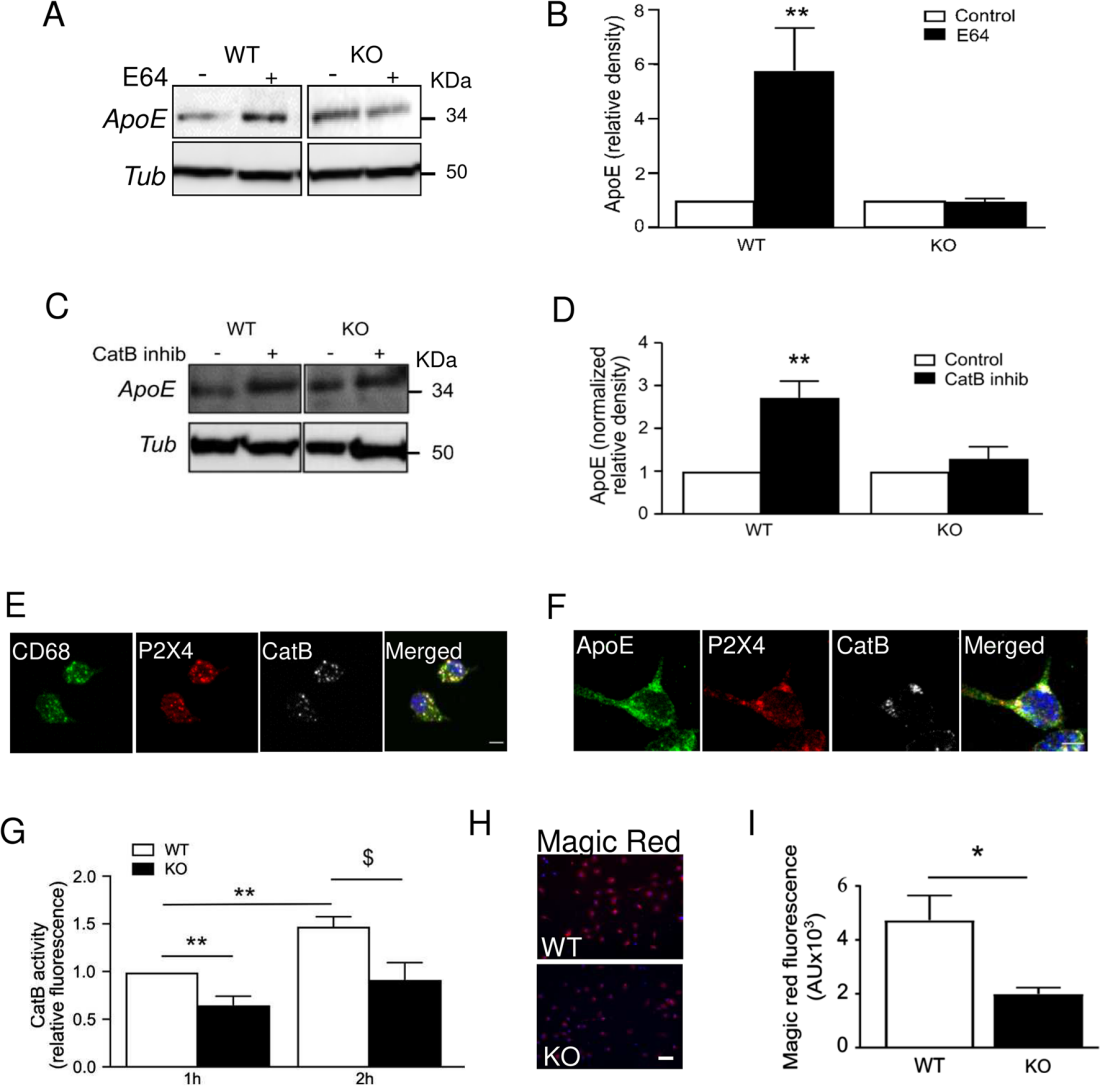
P2X4 regulates cathepsin B-dependent ApoE degradation. **A**, **B** Comparison of treatment with E64, a pharmacological inhibitor of the cysteine proteases, on ApoE expression in BMDM culture of WT and P2X4 KO mice. **A** Representative Western blot of ApoE in the supernatant of WT and P2X4 KO BMDM after incubation with 10 µM E64 overnight. **B** Quantitative analysis of Western blots shows that E64 induced a strong increase of ApoE in the supernatant of WT but not in P2X4 KO BMDM. *N* = 5 independent experiments, **p < 0.01, One sample *t* test compared to theoretical value of 1. **C, D** Comparison of treatment Z-Phe-Ala-FMK, a CatB inhibitor, on ApoE expression in BMDM culture of WT and P2X4 mice. **C** Representative Western blot of ApoE in the supernatant of WT and P2X4 KO BMDM after incubation with 20 µM Z-Phe-Ala-FMK overnight. **D** Quantitative analysis shows that inhibition of CatB with Z-Phe-Ala-FMK induces a strong increase of ApoE in the supernatant of WT but not in P2X4 KO BMDM. *N* = 6 experiments**,** **p < 0.01, One sample *t* test compared to theoretical value of 1. **E, F** Co-localization in BMDM of P2X4, ApoE and CatB in CD68-positive compartments. **E** Representative picture of CD68 (green), P2X4 (red) and CatB (white) immunostaining in BMDM cells. Scale bar 5 µm. **F** Representative immunostaining of ApoE (green), P2X4 (red) and CatB (white), DAPI (blue) in BMDM cells. Images are representative of *N* ≥ 3 experiments. Scale bar 5 µm. **G–I** P2X4 regulates CatB activity in BMDM. **G** CatB activity was measured using the cell-permeable fluorogenic CatB substrate Z-RR-AMC. After incubation with 100 µM Z-RR-AMC, fluorescence was read 1 h and 2 h later. A significant increase of the signal is observed in WT macrophages between 1 and 2 h, whereas the activity in KO cells remained unchanged. *N* = 8 experiments, **p < 0.01, One sample *t* test compared to theoretical value of 1 WT(1 h) vs KO (1 h) and WT (1 h) vs WT(2 h), $ *p* < 0.05 Kruskal–Wallis test WT (2 h) vs KO (2 h); KO (1 h) vs KO (2 h) is non-significant. **H** Representative microscopic image of cellular CatB activity in WT and P2X4 KO BMDM using the Magic red cathepsin B kit. A strong signal is observed in WT BMDM as compared to P2X4 KO cells. **I** Quantitative analysis of the magic Red fluorescence using ImageJ. *p < 0.05, *N* = 3 independent cultures, unpaired *t* test. Scale bar 30 µm

A potential explanation for the higher amount of ApoE in P2X4-deficient myeloid cells is that ApoE is degraded, through its interaction with P2X4 in lysosomes. To test this hypothesis, we used the E64 compound, a known inhibitor of lysosomal proteases [36]. WT and KO BMDM were incubated overnight with 10 µM E64, and levels of secreted and cellular ApoE were evaluated by Western blotting. As shown on Fig. 3A and B, in WT BMDM, E64 treatment strongly increased amounts of intracellular ApoE compared to untreated cells (0.44 ± 0.1 vs 0.23 ± 0.1, unnormalized values). In KO cells, E64 treatment had no effect on ApoE (0.92 ± 0.4 vs 0.81 ± 0.3). E64 is a broad-spectrum cysteine protease inhibitor, which targets many different proteases either cytoplasmic or lysosomal. To determine the proteases involved, we therefore tested more specific inhibitors of cysteine proteases. We first evaluated the potential involvement of calpains, a family of mostly cytoplasmic proteases. Overnight pre-treatment of WT and P2X4-deficient BMDM with 10 µM calpain inhibitor III (CI–III), which targets calpains I and II [37], did not induce any significant change in ApoE amounts in either group (WT: 0.42 ± 0.2 vs CI–III: 0.43 ± 0.2; P2X4 KO: 1.1 ± 0.5 vs CI–III: 1.57 ± 0.7) (Sup. Fig. 6A, B). These results were confirmed using Suc-Leu-Leu-Val-Tyr-AMC, a fluorescent substrate of calpain; incubation of BMDM with 100 µM Suc-Leu-Leu-Val-Tyr-AMC showed no difference in fluorescence signal between genotypes (Sup. Fig. 6C). These results indicate that calpains were not involved in ApoE degradation.

We next tested whether CatB, a cysteine protease highly expressed in lysosomes, could be involved in ApoE degradation [35]. Pre-incubation of WT BMDM with 20 µM of CatB inhibitor [38] overnight strongly enhanced amounts of ApoE (0.15 ± 0.1 vs 0.40 ± 0.2, unnormalized values) while having no effect on P2X4-deficient BMDM (0.44 ± 0.2 vs 0.46 ± 0.1, unnormalized values) (Fig. 3C, D). No effect was observed using specific cathepsin L or cathepsin S inhibitors (data not shown). Triple immunostaining of P2X4, CD68 and CatB revealed a strong co-localization of the three proteins, indicating that P2X4 and CatB are both present in lysosomes (Fig. 3E). A similar co-localization was observed for P2X4, CatB and ApoE (Fig. 3F). Specificity of the CatB antibody was verified by immunostaining and Western blot in CatB-deficient BMDM (Sup. Fig. 7A, B). We analyzed whether P2X4 deletion could alter the enzymatic activity of CatB. We first measured CatB activity using the specific CatB substrate ZZ-RR-AMC, which becomes fluorescent upon cleavage. WT and P2X4-deficient BMDM cells were incubated with 100 µM substrate for either 1 or 2 h and end point fluorescence was measured. As shown in Fig. 3G, fluorescence was significantly higher in WT cells compared to P2X4-deficient BMDM, suggesting that CatB activity is reduced in these cells. These results were further confirmed with Magic Red assay, a cell-permeant CatB substrate whose fluorescence increases upon cleavage. Figure 3H, I shows that after incubation with Magic Red substrate, fluorescence was higher in WT compared to KO cells (47,301 ± 9238 vs 19,969 ± 2357, respectively, fluorescence arbitrary units). This lower CatB activity in P2X4-deleted cells was not due to an impaired expression of the enzyme since both WT and P2X4-deficient cells display similar amounts of CatB (Sup. Fig. 7C). Following our hypothesis that CatB controls the degradation of ApoE, we tested whether BMDM from CatB-deficient mice would express higher level of ApoE. As shown in Sup. Fig. 8, a higher amount of ApoE in CatB-deficient BMDM compare to WT was observed, supporting the role of CatB on ApoE degradation.

### P2X4 is predominantly expressed in plaque-associated microglia

**Fig. 4:**
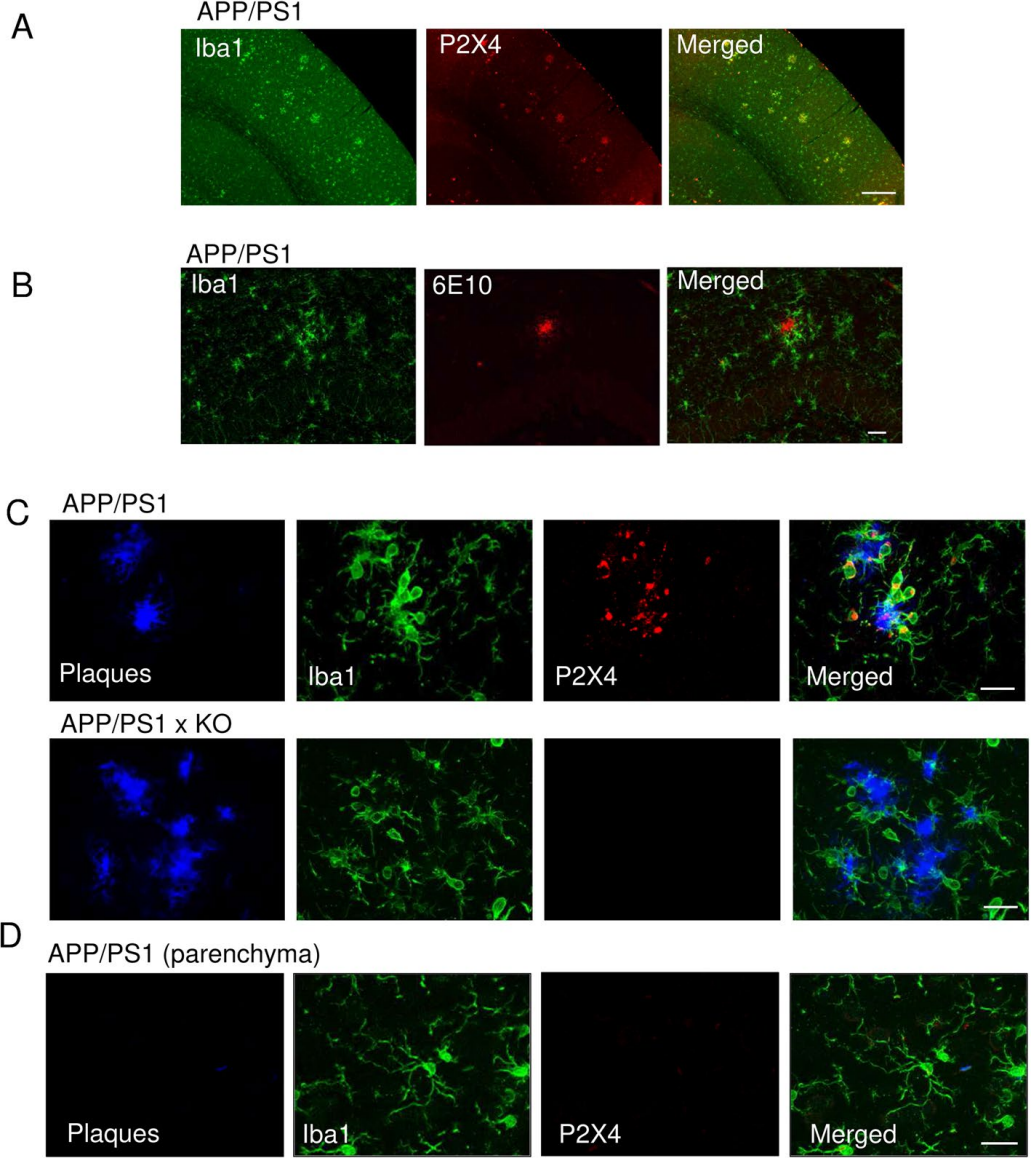
P2X4 is specifically expressed in plaque-associated microglia in mice brain. **A** Representative low magnification pictures of P2X4 (red) and Iba1 (green) immunostaining in the cortex of 12-month APP/PS1 mice. Scale bar 200 µm. **B** Iba1 clusters surround 6E10 positive immunostaining corresponding to amyloid plaques. Scale bar 30 µm. **C** High magnification of P2X4 (red) Iba1 (green) immunostaining at the vicinity of amyloid plaques (Amylo Glo staining, blue) in the cortex of 12-month APP/PS1 mice *(top)* and APP/PS1xKO mice *(bottom)*. Note the specific intracellular localization of P2X4 in microglia clustered around amyloid deposit. Scale bar 20 µm. **D** Representative immunofluorescence in APP/PS1 mice showing that parenchymal microglia (Iba1, green) do not express P2X4 (red) in region with no amyloid deposit (Amylo Glo staining, blue). All images are representative of *N* ≥ 6 independent experiments, *n* ≥ 6 mice for each genotype. Scale bar 20 µm

In pathological conditions such as neuropathic pain or epilepsy, P2X4 receptors are de novo expressed in reactive microglia where they contribute to inflammatory response and neuronal hyperexcitability. The interaction between P2X4 and ApoE, a major risk factor in AD, prompted us to investigate the potential role of P2X4 in AD. P2X4 expression was analyzed in APP/PS1 mice and APP/PS1xP2X4 KO mice (call APP/PS1xKO thereafter). In the cortex of 12-month-old APP/PS1 mice, P2X4 immunostaining co-localizes with that of Iba1, a specific microglial maker in localized patches (Fig. 4A). Co-staining with Iba1 and 6E10 reveals that microglia form cluster around amyloid plaques (Fig. 4B). At higher magnification, triple staining of plaques, microglia and P2X4 shows that in AD brain, P2X4 is specifically expressed in reactive microglial cells clustered around amyloid plaques (Fig. 4C top panel), presumably the so-called disease-associated microglia [7, 39]. P2X4 immunostaining clearly shows an intracellular localization of the protein in microglia, while in APP/PS1xKO, P2X4 immunostaining was absent (Fig. 4C middle panel). In the parenchyma, in regions were plaque are barely present, no obvious P2X4 staining could be observed in microglia (Fig. 4C bottom panel).

### P2X4 regulates ApoE degradation in APP/PS1 mice

**Fig. 5:**
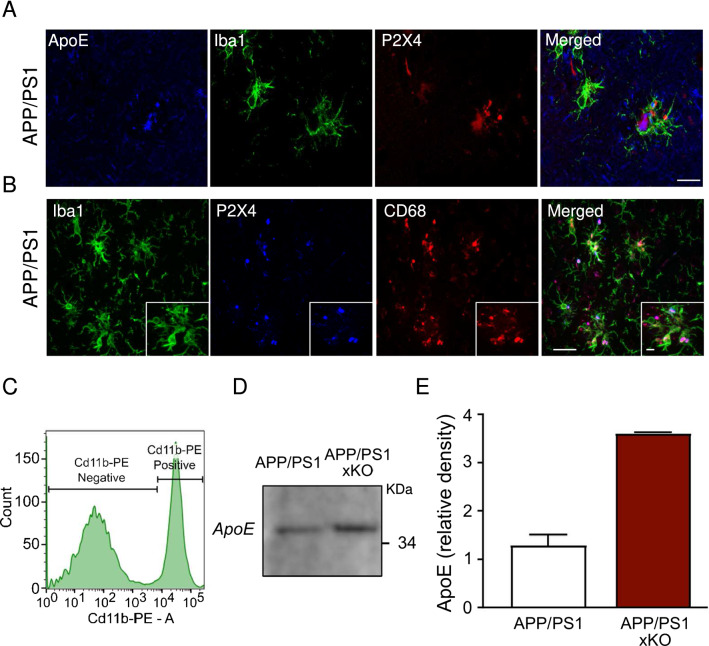
Increased ApoE in microglia from APP/PS1xKO mice. **A** Immunofluorescence of ApoE (blue), Iba1 (green) and P2X4 (red) in APP/PS1 mice cortex. Scale bar 10 µm. **B** Immunofluorescence of Iba1 (green), P2X4 (Blue) and CD68 (red) in APP/PS1 mice cortex showing the expression of P2X4 in microglial lysosomes. Inset: higher magnification fields. All images are representative of *N* ≥ 6 experiments, n ≥ 6 mice. Scale bars 50 µm and 5 µm for high magnification. **C–E** Analysis of ApoE expression in FACS-sorted microglia from APP/PS1 and APP/PS1xKO mice. **C** Microglia were sorted based on CD11b-PE positive selection. **D** Representative Western blot of ApoE from APP/PS1 and APP/PS1xKO FACS-sorted cortical microglia. **E** Quantitative analysis of signals presented in C shows an increase in ApoE in APP/PS1xKO mice relative to APP/PS1 mice. *N* = 2 independent experiments, *n* = 2 mice per group

We next investigated whether in APP/PS1 mice, microglial P2X4 is also prone to regulate ApoE degradation as demonstrated in vitro. First, we analyzed localization of ApoE and P2X4 in 12-month-old APP/PS1 mice. Triple cortical co-immunostaining of ApoE, P2X4 and Iba1 revealed that in APP/PS1 mice, P2X4 co-localizes with ApoE in microglia that are clustered around plaques (Fig. 5A). Furthermore, in reactive microglia P2X4 co-localizes with CD68 + vesicles **(**Fig. 5B**)**, supporting that P2X4 and ApoE interact in lysosome of reactive microglia.

We next quantified by Western blot ApoE from FACS-sorted CD11b + microglia from APP/PS1 and APP/PS1xKO mice (Fig. 5C–E). Confirming in vitro findings, results show that the amount of ApoE was increased in purified microglia from APP/PS1xKO compared to APP/PS1 (0.36 ± 0.001 vs 0.13 ± 0.02, respectively).

### P2X4 deletion reduces sAβ content in cortex of APP/PS1 mice

**Fig. 6:**
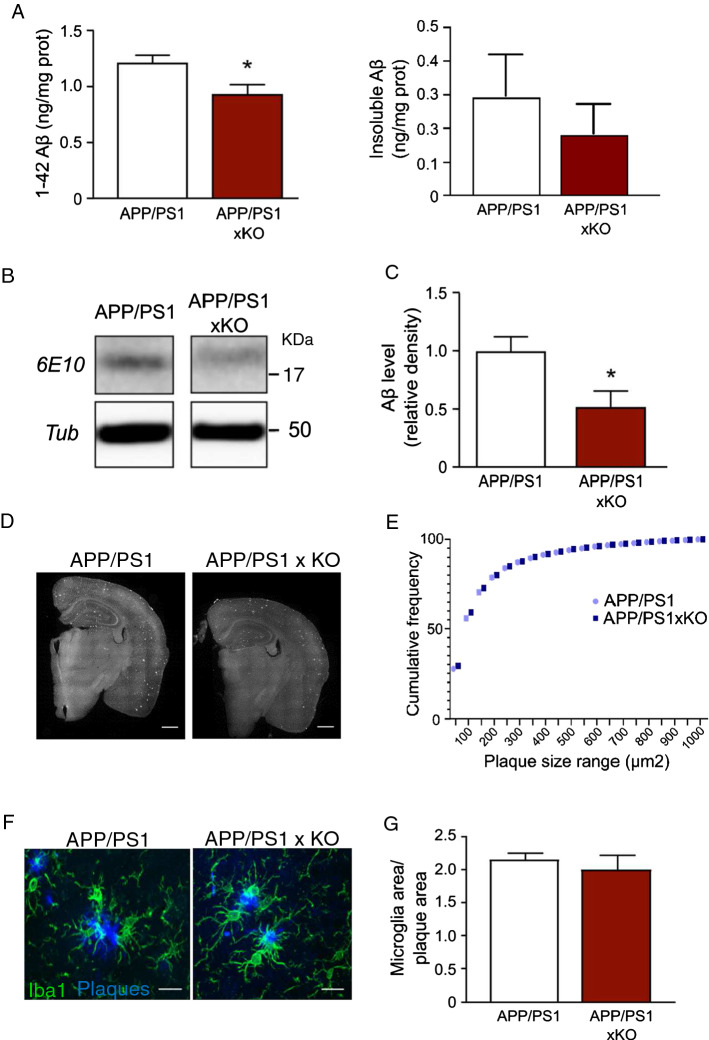
Deletion of P2X4 reduces soluble Aß species but does not affect amyloid plaques density. **A** ELISA quantification of soluble (right panel) and insoluble (left panel) Aβ1–42 peptides in the cortex of APP/PS1 and APP/PS1xKO mice. A significant decrease of the concentration of sAß is observed in APP/PS1xKO mice, compared to APP/PS1 mice. Insoluble Aß peptide is unchanged between the two genotypes. *N* = 2 independent experiments, *n* = 6–7 mice per group. *p < 0.05, unpaired *t* test. **B** Representative Western blot of Aß peptide detected with the 6E10 antibody from cortex extracts of APP/PS1 and APP/PS1xKO mice. **C** Quantitative analysis of Western blots presented in (**B**). A significant decrease of the Aβ peptide amount is observed in APP/PS1xKO mice. *N* = 3 independent experiments, *n* = 7 mice per group, *p < 0.05, unpaired *t* test. **D** Representative images of Thioflavine T staining in APP/PS1 and APP/PS1xKO brain. *N* ≥ 6–7 independent experiments. Scale bar 700 µm. **E** Cumulative frequency of the range size of amyloid plaques. There is no obvious difference in the number of plaques nor in their size between APP/PS1 and APP/PS1xKO; *n* = 11 mice per group. **F** and **G** Analysis of microglial clustering around amyloid plaque between in the cortex of APP/PS1 and APP/PS1xKO mice. **F** Representative image of microglia clustering around plaques. Amyloid plaques are stained with AmyloGlo (blue) and microglial is stained with Iba1 (green), *n* = 11 mice. Scale bar 20 µm. **G** Quantification of the area covered by microglia surrounding amyloid plaques. The ratio of the surface occupied by microglia over the surface of the plaque is expressed for both APP/PS1 and APP/PS1xKO mice. *n* = 11 mice per group, unpaired t-test

ApoE is thought to be involved in plaque formation and clearance of Aß peptide and memory performance deficits in AD have been linked to soluble small Aß oligomers [40]. We thus analyzed whether the level of the soluble Aß (sAß) peptide was affected by P2X4 deletion in APP/PS1. Quantification of sAß_1–42_ peptide by ELISA in the cortex of APP/PS1xKO and APP/PS1 showed a significant lower amount of soluble Aβ in the absence of P2X4 (0.937 ± 0.083 ng/mg vs 1.216 ± 0.066 ng/mg, respectively), while insoluble Aß species were not significantly different between the two genotypes **(**Fig. 6A**)**. Consistent with these findings Western blot analysis of cortical extracts confirmed that the amount of low-molecular-weight sAβ was reduced in APP/PS1xKO compared to APP/PS1 mice (Fig. 6B, C, 0.52 ± 0.1 vs 1 ± 0.1, respectively). We also assessed whether the deletion of P2X4 could affect amyloid plaque load in APP/PS1 mice. Number of plaques, their average size and the number of microglia associated with plaques were quantified in the cortex of 12-month APP/PS1 and APP/PS1xKO after AmyloGlo staining **(**Fig. 6D**)**. As shown in Fig. 6E, in APP/PS1 mice, P2X4 deletion did not alter density of plaques, their average seize nor the average number of microglia clustered around plaques (Fig. 6F, G).

### P2rX4 deletion reverses memory deficits in APP/PS1 mice

**Fig. 7:**
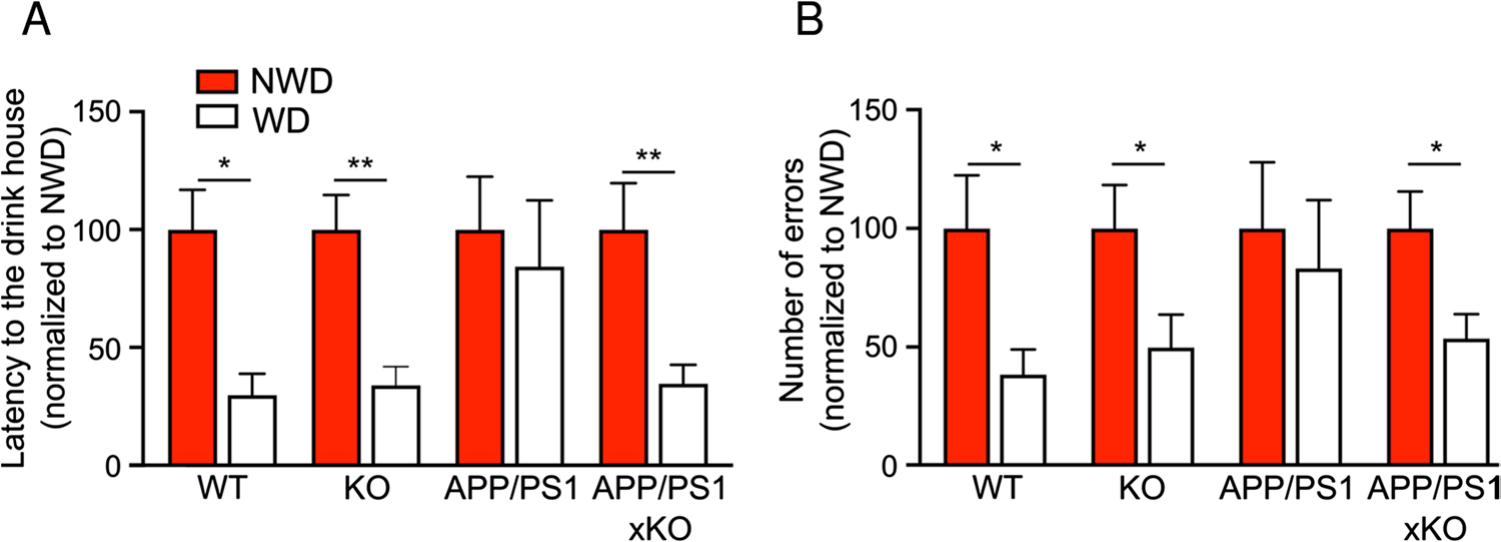
*P2rX4* deletion reverses memory deficit in APP/PS1 mice in the Hamlet test. **A** Latency to locate the drink house 15 h after water deprivation in the Hamlet test. WT and KO mice present reduced latency to the drink house, whereas no difference was observed between non-water deprived (NWD) and water-deprived (WD) conditions in APP/PS1 mice. APP/PS1xKO water-deprived mice present significant reduction of the latency, indicating that mice have retained the location of the drink house. **B** Number of errors before entering the drink house. WT and KO mice present reduced number of errors, whereas no difference was observed between non-water and water-deprived condition in APP/PS1 mice. APP/PS1xKO water-deprived mice present significant reduction of the number of errors. *N* = 3 independent experiments, *n* = 8–11 mice per group. *p < 0.05, **p < 0.01, Mann–Whitney test, WD vs NWD for each genotype

Alteration of cognitive performance is a hallmark of AD and particularly spatio-temporal disorientation is an early sign of the disease in human. APP/PS1 mice display decline of cognitive performance in a variety of learning behavioral tests meant to assess spatial memory [41, 42]. We therefor address whether the upregulation of microglial P2X4 in APP/PS1 mice has any impact on memory performance. First, we used the Hamlet test [28, 29] to assess topographical memory in the different groups of animals. 72 h after the last training session, mice were water deprived (WD) for 15 h. The probe test was performed by placing mice in the central agora for 10 min. Latency and number of errors to reach the drink house were analyzed. A second probe test was repeated the following day, animals being once again placed in the apparatus but in non-water deprived (NWD) condition. Both WD-WT and WD-KO mice showed a significant shorter latency to reach the drink house as compared to NWD condition, signing proper memory (Fig. 7, left panel, WD-WT: 24.4 ± 7.3 s; NWD-WT: 81.5 ± 1.7 s; WD-KO: 13.9 ± 3.2 s; NWD-KO: 40.9 ± 6 s). As expected, performances of WD-APP/PS1 mice were not different from that of NWD-APP/PS1 (WD-APP/PS1: 73.5 ± 24.5 s and NWD-APP/PS1: 87.1 ± 19.5 s), suggesting impaired memory. Remarkably, learning deficits observed in APP/PS1 mice were reverted in APP/PS1xKO mice (WD-APP/PS1xKO: 39 ± 9 s and NWD-APP/PS1xKO: 112.4 ± 22.1 s) and performances were found similar to both WT and KO animals.

Similar data were found when addressing the total number of errors. The later was reduced in both WD-WT and KO mice compared to NWD animals (WD-WT: 14.1 ± 3.9 vs NWD-WT: 36.8 ± 8.2; WD-KO: 11.1 ± 3.1 vs NWD-KO: 22.3 ± 4.1). This difference was absent in WD-APP/PS1 mice (WD: 33.5 ± 11.6 vs NWD: 40.3 ± 11.2), but readily observed in WD-APP/PS1x KO (WD: 23 ± 4.5 vs NWD: 43.7 ± 6.8) (Fig. 7, right panel). The Morris Water maze test also indicated that although the learning curve was not different between genotypes, only APP/PS1 mice showed altered memory retention (Sup. Fig. 9A, B, C). Locomotor activity of the different genotypes analyzed in the open field task indicated that both APP/PS1 and APP/PS1xKO mice presented a tendency to higher locomotion compared to WT and P2X4 KO, ruling out that the alteration observed in the Hamlet test could relate to mobility deficits (Sup. Fig. 9D). These results indicated that invalidation of P2X4 rescued memory deficits observed in APP/PS1 mice and support a role of P2X4 likely expressed by reactive microglia in cognitive alteration associated with AD.

### P2X4 and ApoE are co-expressed in plaque-associated microglia in human AD brain

**Fig. 8:**
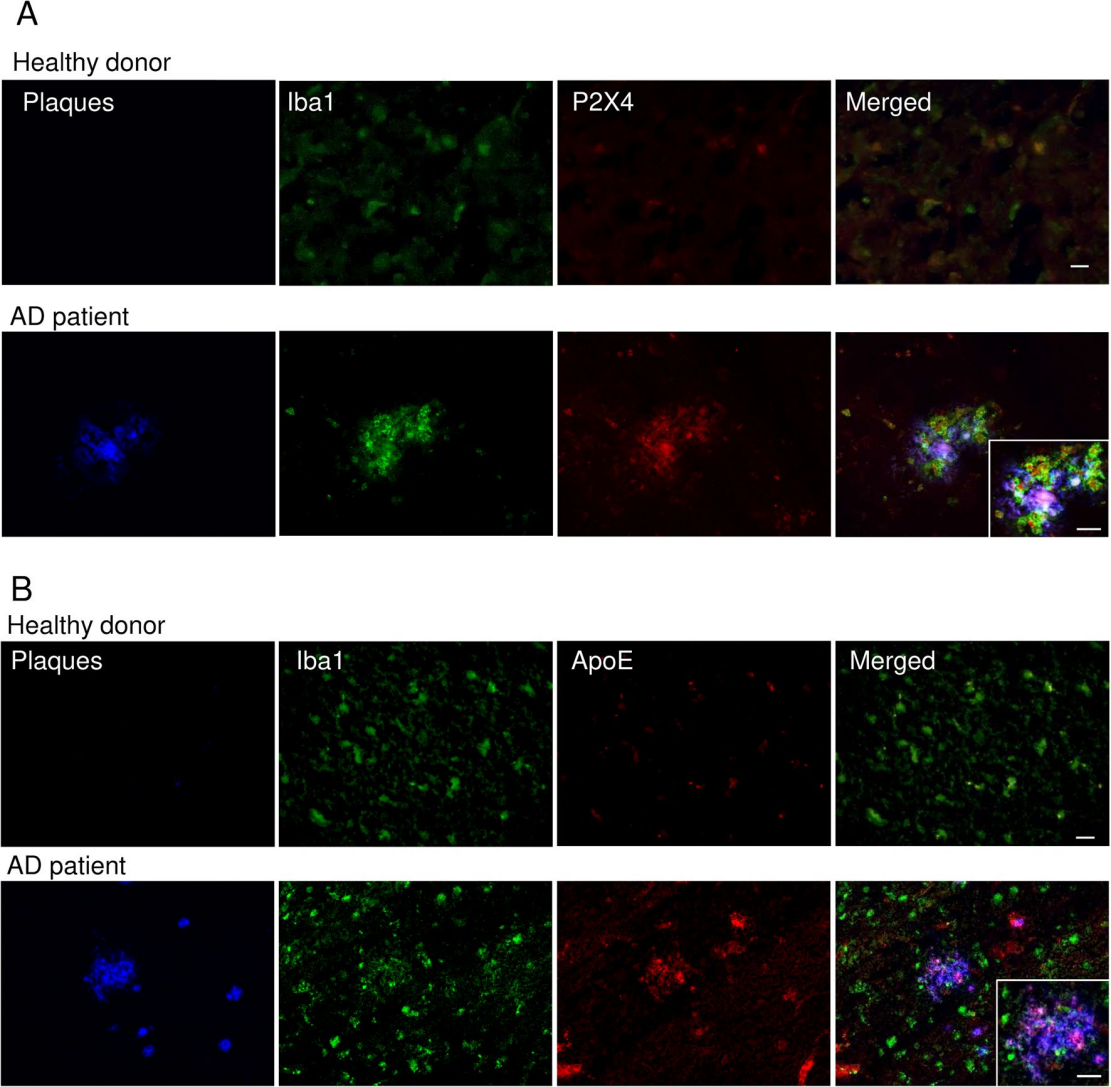
Expression of P2X4 in AD human brain. **A** Representative pictures of cortical brain slices from AD patients and healthy control labeled with AmyloGlo (blue, amyloid plaques), Iba1 (green) and P2X4 (red). P2X4 staining co-localizes with Iba1 in regions of dense amyloid plaque staining, supporting that microglia clustered around amyloid deposit specifically express P2X4. In healthy control brain, P2X4 staining is dim and does not co-localizes with that of Iba1, indicating that P2X4 is not expressed in non-reactive microglia. Inset: higher magnification field. Scale bars 10 µm and 10 µm (inset). **B** Representative pictures of cortical brain slices from healthy donor and AD patients labeled with AmyloGlo (blue, amyloid plaques), Iba1 (green) and ApoE (red) showing an increased expression of ApoE in human microglia clustered around amyloid deposit. Inset: higher magnification field. Scale bars 30 µm and 10 µm (inset). *N* = 3–6 patients per condition

Using cortices slices from AD human patients, immunohistochemistry reveals a strong P2X4 immunostaining co-localized with Iba1 and amyloid plaques staining, while in healthy brain, P2X4 staining was almost absent (Fig. 8A). Similarly, a clear co-localization of amyloid plaques, ApoE and Iba1 was also observed in brain of AD patient, whereas in healthy brain, ApoE staining was slight (Fig. 8B). Together these results support that in human AD brain, microglial P2X4 could also regulate ApoE degradation.

## Discussion

P2X4 receptor expression is up-regulated in reactive microglia associated with diverse neuropathological conditions such as neuropathic pain, *status epilepticus* or multiple sclerosis [22]. Their activation in reactive microglia generally promotes deleterious effects such as hyperexcitability or inflammation [43]. Yet, in multiple sclerosis, P2X4 expression has beneficial effect by increasing myelin phagocytosis and favoring remyelination [44]. In this study, we investigated to what extend P2X4 contributes to microglial functions in Alzheimer’s disease.

Using a proteomic approach, we identified a specific interaction between ApoE and P2X4 in macrophages and microglia, and demonstrated that this interaction leads to a CatB-dependent degradation of ApoE. In brain of APP/PS1 mice, P2X4 receptors and ApoE are specifically expressed in so-called disease-associated microglia, a subpopulation of reactive microglia clustered at the vicinity of amyloid plaques. In microglia from APP/PS1 invalidated for the *P2rX4* gene, a higher amount of ApoE was observed, which correlated with a reduction of small soluble Aß aggregates while there was no major difference in amyloid plaque load. *P2rX4* deletion in APP/PS1 reversed spatial and topographical memory deficits associated with the APP/PS1 genotype to what is observed in wild-type mice. Finally, a similar pattern of expression of P2X4 and ApoE in microglia was also observed in post mortem human brain from AD patients.

### P2X4 interact with ApoE and mediates its degradation

P2X4 receptors are mainly located in the endosomal/lysosomal network, which structural dysregulation in AD could promote abnormal APP processing [45]. Using a proteomic strategy based on intracellular organelle enrichment [46, 47], we identified ApoE as a specific P2X4 interacting protein in both macrophage and microglia cells. Both proteins co-localize in intracellular CD68-positive compartments, likely endo-lysosomes, and deletion of *P2rX4* results in higher amounts of intracellular and secreted ApoE. Our findings support that P2X4 drives CatB-dependent ApoE degradation since, (i) inhibition of CatB enhances extracellular amounts of ApoE, an effect that is not observed in P2X4-deficient cells, and, (ii) CatB activity is reduced in P2X4-deficient cells. How P2X4 regulates CatB activity remains unclear. Our data show that in recombinant system, introduction of an ATP-binding site blocking mutation in P2X4 does not alter ApoE degradation, suggesting that P2X4 activity is not required to promote ApoE degradation. Furthermore, while in the endo-lysosomal pathway ATP-binding region of P2X4 faces the organelle’s lumen and its high ATP concentrations, acidic pH reduces P2X4 affinity for ATP [48] preventing the receptor activation. Even though alkalinization of lysosome may lead to P2X4 activation as previously shown [49], such an alkalinization would also lead to a decrease of CatB activity [50], which *in fine* would reduce ApoE degradation. In addition, using the fluorescent Lysosensor^™^, we did not observe any variation of intra-lysosomal pH in P2X4-deficient cells, further supporting that regulation of CatB activity by P2X4 is independent of pH variation (data not shown). Yet, in the context of AD where dysregulation of lysosomal pH is well documented [51], we cannot totally exclude that an activity of lysosomal P2X4 could contribute to ApoE degradation.

A key feature of P2X4 is its de novo expression in reactive microglia in diverse pathological conditions. Our data show that in APP/PS1 mice, P2X4 is almost exclusively expressed in plaque-associated microglia, but not in parenchymal microglial away from plaques nor in neurons. It is likely that P2X4 belongs to the so-called DAM [39], a specific microglial population that is characterized by the specific expression of subset of genes, including several known AD risk factor such as *Trem2* and *ApoE*. Our data show a strong co-localization of P2X4 and ApoE in plaque-associated microglia, in both mice and human AD patients, and in a recent study using mass spectrometry to identify deregulated proteins in microglia, an increase of P2X4 was observed in two mouse models of AD (APP/PS1 and APP-NL-G-F) [52]. While a clear de novo P2X4 protein expression is observed in reactive microglia, *P2rX4* gene has not been identified as deregulated in the various high throughput global or single cell genomics studies of reactive microglia [7, 39, 53]. However, using spatially defined transcriptome analysis of plaque-associated microglia, we found a fourfold increase of mP2X4 mRNA in DAM, further supporting a specific role of P2X4 in this subpopulation of microglia [54]. This discrepancy between protein and RNA might be due to a regulation of the translation of P2X4 mRNA as previously suggested [55].

### Role for microglial P2X4/ApoE in AD

Our results show that in AD mouse brain, P2X4 is specifically expressed in microglia clustered around plaque that also express ApoE. P2X4 deficiency results in a significantly higher level of microglial ApoE, and of its secreted form. P2X4 deficiency also leads to lower the amount of sAß. A great wealth of studies support a direct role of ApoE on sAß clearance [56]. It is surprising to observe that elevated levels of microglial ApoE correlate with reduced sAß and reverse memory impairment in APP/PS1xP2X4KO mice. Indeed, mouse ApoE is thought to be amylogenic since global knock-out of *ApoE* results is dramatic reduction of Aß peptide deposition as well as neuritic dystrophy [57]. However, the contribution of *ApoE* to AD is complex and could be different depending on the soluble state of Aß or the cell type producing ApoE [58–60]. A recent study demonstrated that microglial-specific inactivation of *ApoE*, beside a slight increase of average plaque size, has only limited repercussion of amyloid burden in the 5xFAD model [61]. Yet, microglial *ApoE* deletion results in an age-dependent reduction of the synaptic markers postsynaptic density protein 95 (PSD95) and synaptophysin, regardless of 5xFAD genotype. Our results show that deletion of P2X4 increases microglial ApoE, reduces sAß and reverses cognitive deficits, further supporting a minimal role of microglial ApoE in amyloid plaque formation but a potential protective function toward synapse integrity.

### *P2rX4* deletion in APP/PS1 mice reverses memory impairment

Both spatial and topological memories were assessed in the Morris water maze, and in the Hamlet maze, a recent behavioral device previously shown to measure spatio-temporal disorientation in mice [29]. In these test, APP/PS1 mice show altered memory performances, which are no longer present in APP/PS1xP2X4KO. If *P2rX4* deletion has been linked to alteration of synaptic plasticity, which could result in spatial memory deficit [24], our data supported that, in both the Hamlet test and water maze test, P2X4-deficient mice do not show learning impairment nor retention deficit. Other cognitive deficits have been reported in P2X4 KO mice [62]; however, these deficits relate to socio-communicative and sensorimotor impairments that are not related to hippocampal functions.

In physiological conditions, P2X4 is expressed at low level in different neuronal populations throughout the brain, but absent from microglial cells [63]. In pathological conditions, P2X4 is expressed de novo in reactive microglia where it contributes for instance to BDNF release, network excitability and inflammatory response [20, 21]. In 12 month-old APP/PS1 mice, our data clearly show a strong expression of P2X4 in reactive plaque-associated microglia (PAM), while in aged match control mice, the expression of the receptor is barely detectable. Increased expression of P2X4 in PAM is further supported by recent transcriptomic data which show that in laser captured plaque-associated microglia, *P2rX4* is expressed more than fourfold compared to microglia from the parenchyma, away from any visible plaque [54]. Although we cannot totally exclude a contribution of neuronal P2X4 receptor, our observations that (i) the receptor is highly expressed in PAM microglia and, (ii) its deletion reverse cognitive deficits of APP/PS1 mice strongly support that microglial P2X4 receptor directly contributes to topographic and spatial memory alterations in AD mice.

P2X4 deletion in APP/PS1 mice does not significantly change the number of amyloid plaques, nor the number of microglia in the parenchyma. Yet in APP/PS1xKO, the amount of hippocampal soluble Aß was significantly reduced compared to APP/PS1 mice, while insoluble fraction was not different between the two genotypes. There are compelling evidence that toxic soluble low-molecular-weight Aß oligomers directly induce synaptic impairment leading to learning deficits [40]. The reduction of the amount of sAß observed in APP/PS1xKO could explain their better cognitive performances compared to APP/PS1 mice. This is further supported by the observation of a reduction of GluN1 in the hippocampal synaptosome fraction of APP/PS1 that is not present in APP/PS1xKO (Sup. Fig. 10). One interpretation is that reduced sAß levels observed in APP/PS1xKO may be sufficient to restore synaptic efficiency by directly regulating synaptic integrity alterations associated with sAß oligomers [64].

Although our results do not directly demonstrate a link between the increase of microglial ApoE in P2X4-deficient mice and the reduction of sAß or the attenuation of memory dysfunctions, the specific upregulation of P2X4 receptors in plaque-associated microglia and its role in ApoE degradation strongly support the involvement of this receptor to AD behavioral deficits. Our observations of a similar co-expression of P2X4 and ApoE in microglia around amyloid plaques in *post mortem* human AD brain support that P2X4 could play similar functions in the human pathology, although AD mice models only partially recapitulate the human disease and mouse and human ApoE differently contribute to the disease [56]. Further experiments will be necessary to investigate this possibility.

Altogether, our data further support an important contribution of microglial P2X4 receptor to brain pathologies such as neuropathic pain, epilepsy or stroke. They also underline a potential protective function of microglial ApoE toward neurons and cognitive performances.

## Supplementary Information

Below is the link to the electronic supplementary material.

Sup figure 1: Example of the gating strategy for the flow cytometry based on detection of CD11b-PE cells. Cells were labeled with a CD11b-PE antibody, discriminated by size and granularity and microglia were then sorted using a laser with a 561 nm excitation wavelength and a 582 nm filter, with a purity above 95%. Sup figure 2: Co-purification of Apolipoprotein E in P2X4-signaling complex of mouse BMDM membrane extracts. Membrane enriched fractions of mouse BMDM were immunoprecipitated with a specific anti-P2X4 antibody. Pulled-down protein complexes were separated by polyacrylamide gel electrophoresis, trypsinized and analyzed by LC MS/MS. Coverage of ApoE (Swiss-Prot P08226) by MS/MS identified peptides is indicated in green. Coverage results from two independent MS/MS experiments. Total coverage is 22.6% of ApoE sequence (84/371 residues). Sup figure 3: Comparison of non-normalized versus normalized relative ApoE levels in BMDM cells. ApoE in BMDM cell lysates from WT and KO mice were analyzed by Western blot and results expressed relative to tubulin. Results were very variable from culture to cultures. (A) Results from normalized experiments. Value obtained from KO sample were normalized to an arbitrary value of 1 attributed to WT samples (B) Same data as in A, except that results are non-normalized experiments. Results are expressed as a ratio of ApoE band density over that of tubulin obtained in lysates. Essentially similar results were obtained when data from the same experiments as in B were normalized to signal obtained in WT condition, albeit with lower variability. N = 4 independent experiments, * p < 0.05, unpaired t-test. Sup figure 4: Comparison of ApoE levels in WT and P2X4 KO microglial primary cultures. ApoE levels in microglial cell lysates were analyzed by Western blot. Data were normalized to signal obtained from WT culture. As in BMDM, significant increase of ApoE is observed in P2X4 KO microglia cultures. N = 5 independent experiments, * p < 0.05, unpaired t-test. Sup figure 5: ApoE transcription is not affected in P2X4 KO BMDM. BMDM from WT and P2X4 KO mice were stimulated or not with 100 ng of LPS overnight. After RNA extraction, expression of ApoE mRNA was quantified by RT-qPCR. No difference in ApoE mRNA levels could be observed between the different conditions. N = 3 independent experiments, two-way ANOVA. Sup figure 6: Calpains are not involved in ApoE degradation. (A) Representative Western blot of ApoE and P2X4 from WT and P2X4 KO BMDM cell lysates, treated or not with 10 µM calpain inhibitor III overnight. (B) Quantification of ApoE signal from A normalized to the WT, no drug condition. N = 3 independent experiments, one-sample Wilcoxon test compared to the theoretical value of 1. (C) Analysis of calpain activity using the fluorogenic calpain substrate Suc-leu-leu-val-tyr AMC (100 µM). Fluorescence was acquired 3 and 4h after addition of the substrate. No difference was observed between WT and KO BMDM cells. N = 3 independent experiments. Sup figure 7: P2rx4 deletion does not affect the expression of CatB. (A and B) Validation of the specificity of the CatB antibody by immunocytochemistry and Western blot. N = 2 independent experiments. Scale bar 5 µm. (C) Western blot analysis of CatB expression in WT and P2X4 KO BMDM cells shows no obvious difference of CatB expression between the two genotypes. N=3 independent experiments. Sup figure 8: Increase of ApoE in CatB KO BMDM. (A) Representative western blot of ApoE from WT and CatB KO BMDM cell lysates. (B) Quantification of ApoE signal presented in A. N = 2 independent experiments. Sup figure 9: Increased memory performances of APP/PS1xKO mice in the Morris water maze. (A) Comparison of the learning curve of WT, P2X4 KO, APP/PS1 and APP/PS1xKO. The time spent to reach the platform for each day of training is reported for each genotype. Compared to WT, all other genotypes seem to learn slower but differences are not statistically different from WT, Two-way ANOVA. (B) Retention test analysis. 48h after the last day of training, time spent in the target quadrant was measured and compared to the theorical value of 25%. Only APP/PS1 mice show no statistical difference, whereas all other genotypes including APP/PS1xKO spend significatively more time in the targeted quadrant. *** p<0.001, one sample Wilcoxon test. (C) Representative swim paths during the retention test for the 4 genotypes. (D) Activity of mice in the open field test. Total distance traveled by mice in the whole arena during 10 min recording. (n = 16, 10, 20 and 18 mice for WT, APP/PS1, KO and APP/PS1xKO respectively, * p< 0.05, One-way ANOVA). Sup figure 10: P2X4 deletion reverses reduction of GluN1a expression observed in APP/PS1 mice. Quantification by Western blot of GluN1a from WT, P2X4-/-, APP/PS1 and APP/PS1xKO cortex extract. Data were normalized to actin. GluN1a expression is significantly reduced in APP/PS1 compared to WT, P2X4-/-. Deletion of P2X4 in APP/PS1 reverses the down regulation of GLUN1a. One-Way ANOVA, Tukey's multiple comparisons test. * p < 0.05, *** p < 0.001, NS non-significant. N=3 experiments. Supplementary file1 (PDF 629 KB)

## Data Availability

Materials are available from the corresponding author on reasonable request.

## Abbreviations

Aß: Amyloid-ß
AD: Alzheimer’s disease
ATP: Adenosine triphosphate
ALS: Amyotrophic lateral sclerosis
ApoE: Apolipoprotein E
APP: Amyloid-beta A4 protein
BMDM: Bone-marrow-derived macrophage
CatB: Cathepsin B
CNS: Central nervous system
DAM: Disease-associated microglia
DMEM: Dulbecco’s Modified Eagle Medium
ELISA: Enzyme-linked immunosorbent assay
FACS: Fluorescence-activated cell sorting
FBS: Fetal bovine serum
GWAS: Genome-wide association studies
HBSS: Hank’s balanced salt solution
Iba1: Ionized calcium-binding adapter molecule 1
KO: Knock-out
NWD: Non-water deprived
PAM: Plaque-associated microglia
PFA: Paraformaldehyde
PS1: Presenilin 1
PSD95: Postsynaptic density protein 95
RT-qPCR: Reverse transcription-quantitative polymerase chain reaction
Trem2: Triggering receptor expressed on myeloid cells 2
WD: Water-deprived
WT: Wild-type
