# Supplementary material for: Microglial P2X4 receptors promote ApoE degradation and contribute to memory deficits in Alzheimer’s disease
Source: Cell Mol Life Sci. 2023 May 5;80(5):138. doi: 10.1007/s00018-023-04784-x (PMC10163120; doi:10.1007/s00018-023-04784-x)
Supplement: Supplementary file 1 — Sup figure 1: Example of the gating strategy for the flow cytometry based on detection of CD11b-PE cells. Cells were labeled with a CD11b-PE antibody, discriminated by size and granularity and microglia were then sorted using a laser with a 561 nm excitation wavelength and a 582 nm filter, with a purity above 95%. Sup figure 2: Co-purification of Apolipoprotein E in P2X4-signaling complex of mouse BMDM membrane extracts. Membrane enriched fractions of mouse BMDM were immunoprecipitated with a specific anti-P2X4 antibody. Pulled-down protein complexes were separated by polyacrylamide gel electrophoresis, trypsinized and analyzed by LC MS/MS. Coverage of ApoE (Swiss-Prot P08226) by MS/MS identified peptides is indicated in green. Coverage results from two independent MS/MS experiments. Total coverage is 22.6% of ApoE sequence (84/371 residues). Sup figure 3: Comparison of non-normalized versus normalized relative ApoE levels in BMDM cells. ApoE in BMDM cell lysates from WT and KO mice were analyzed by Western blot and results expressed relative to tubulin. Results were very variable from culture to cultures. (A) Results from normalized experiments. Value obtained from KO sample were normalized to an arbitrary value of 1 attributed to WT samples (B) Same data as in A, except that results are non-normalized experiments. Results are expressed as a ratio of ApoE band density over that of tubulin obtained in lysates. Essentially similar results were obtained when data from the same experiments as in B were normalized to signal obtained in WT condition, albeit with lower variability. N = 4 independent experiments, * p < 0.05, unpaired t-test. Sup figure 4: Comparison of ApoE levels in WT and P2X4 KO microglial primary cultures. ApoE levels in microglial cell lysates were analyzed by Western blot. Data were normalized to signal obtained from WT culture. As in BMDM, significant increase of ApoE is observed in P2X4 KO microglia cultures. N = 5 independent experiments, * p < 0. [file 18_2023_4784_MOESM1_ESM.pdf]

Sup Fig 1

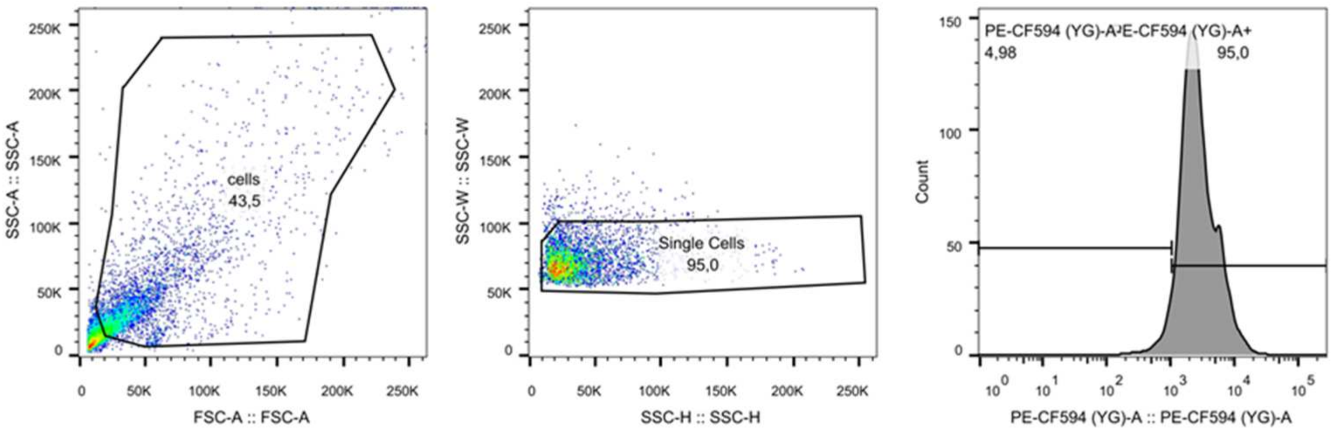
cells
43,5
SSC-A :: SSC-A
FSC-A :: FSC-A
Single Cells
95,0
SSC-W :: SSC-W
SSC-H :: SSC-H
PE-CF594 (YG)-A-PE-CF594 (YG)-A+
4,98
95,0
Count
PE-CF594 (YG)-A :: PE-CF594 (YG)-A

Sup Fig 2

```
MKALWAVLLV TLLTGCLAEG EPEVTDQLEW QSNQPWEQAL NRFWDYLRWV
QTLSDQVQEE LQSSQVTQEL TALMEDTMTE VKAYKKELEE QLGPVAEETR
ARLGKEVQAA QARLGADMED LRNRLGQYRN EVHTMLGQST EEIRARLSTH
LRKMRKRLMR DAEDLQKRLA VYKAGAREGA ERGVSAIRER LGPLVEQGRQ
RTANLGAGAA QPLRDRAQAF GDRIRGRLEE VGNQARDRLE EVREHMEEVR
SKMEEQTQQI RLQAEIFQAR LKGWFEPIVE DMHRQWANLM EKIQASVATN
PIITPVAQEN Q
```

Sup Fig 3

A

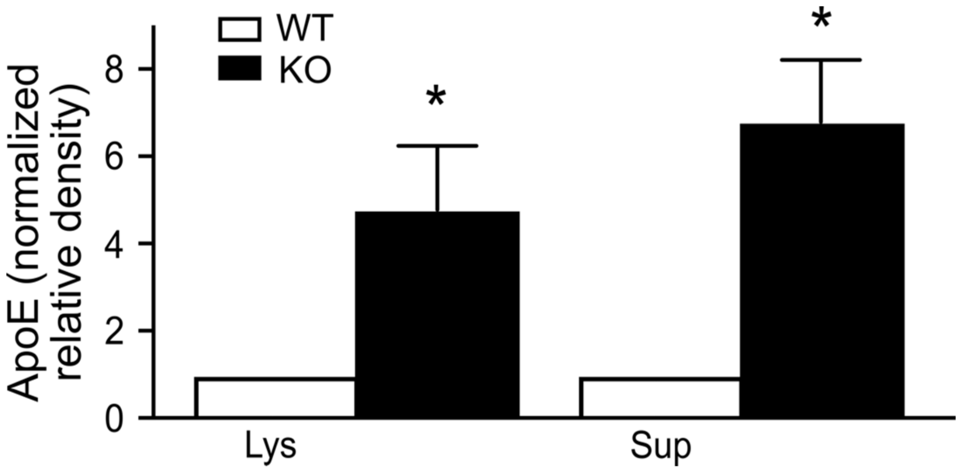
WT
KO
ApoE (normalized relative density)
0
2
4
6
8
*
*
Lys
Sup

B

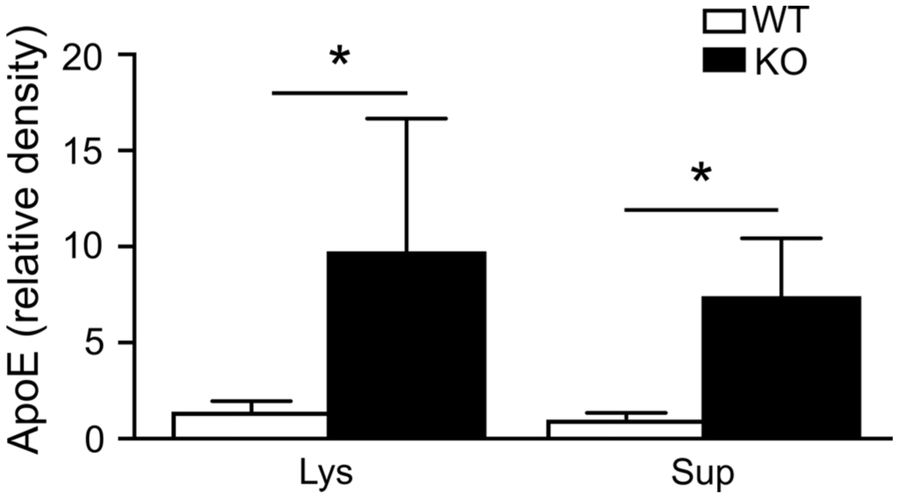
WT
KO
ApoE (relative density)
0
5
10
15
20
*
*
Lys
Sup

Sup Fig 4

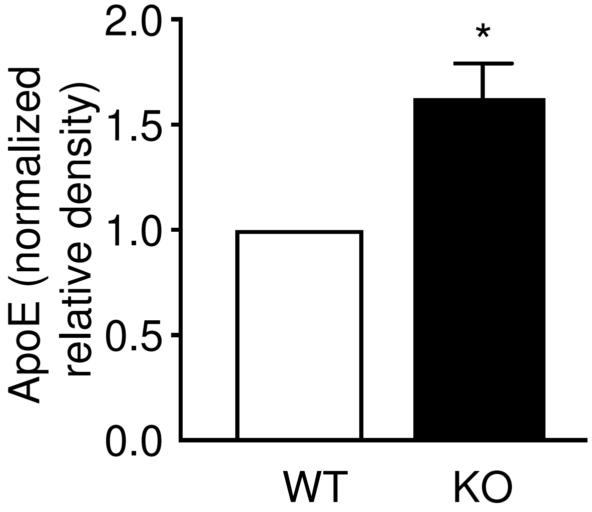

Sup Fig 5

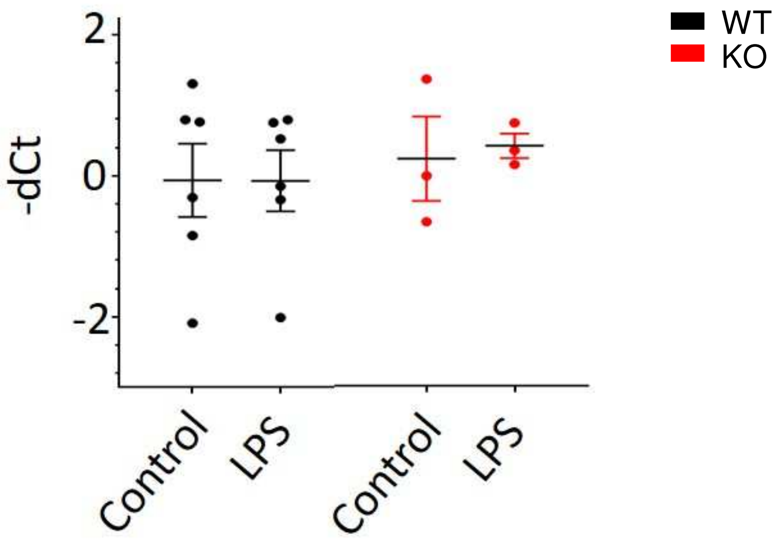

Sup Fig 6

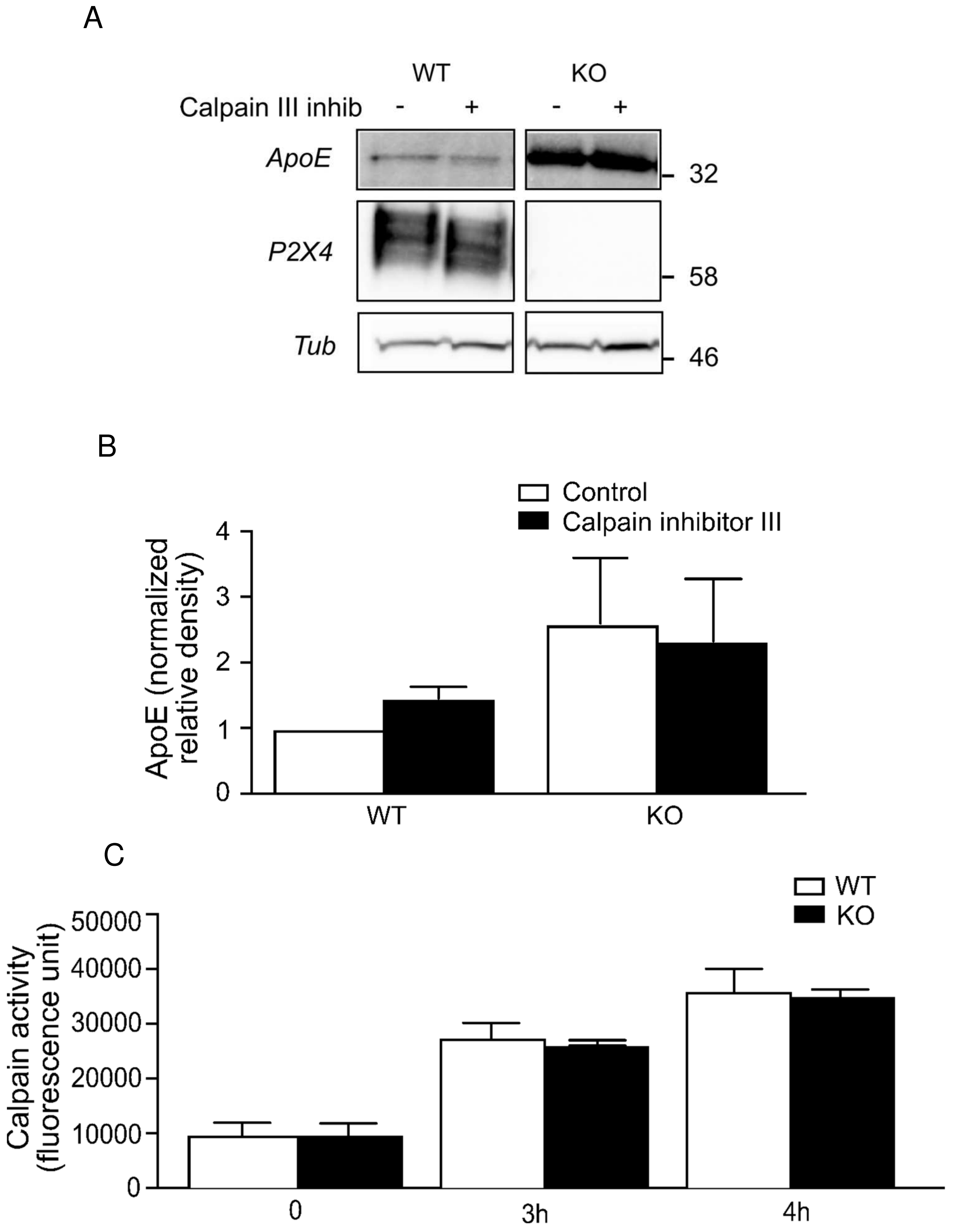
A
WT
KO
Calpain III inhib
-
+
-
+
ApoE
P2X4
Tub
32
58
46
B
Control
Calpain inhibitor III
ApoE (normalized relative density)
0
1
2
3
4
WT
KO
C
WT
KO
Calpain activity (fluorescence unit)
0
10000
20000
30000
40000
50000
0
3h
4h

Sup Fig 7

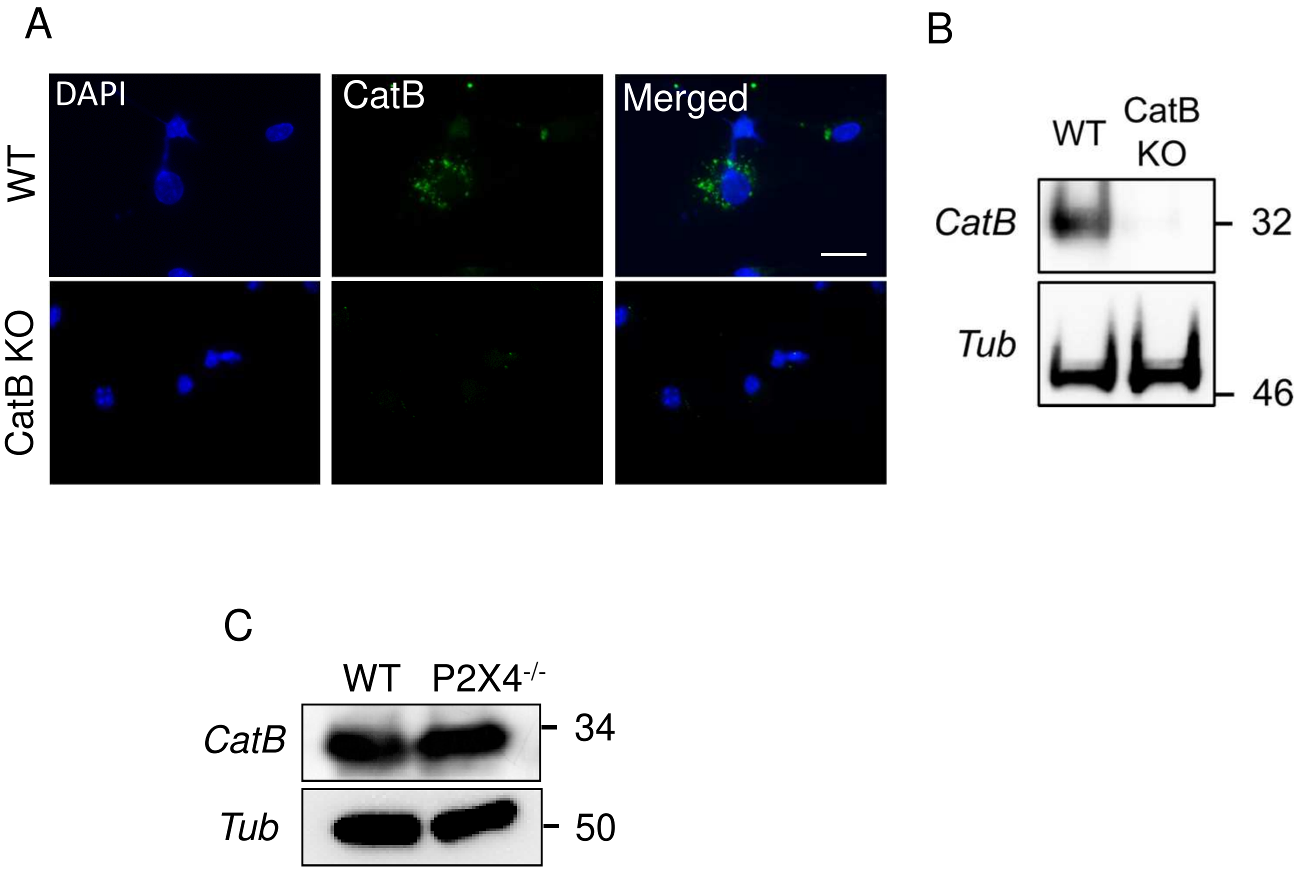
A
DAPI
CatB
Merged
WT
CatB KO
B
WT
CatB KO
CatB
Tub
32
46
C
WT
P2X4-/-
CatB
Tub
34
50

Sup Fig 8

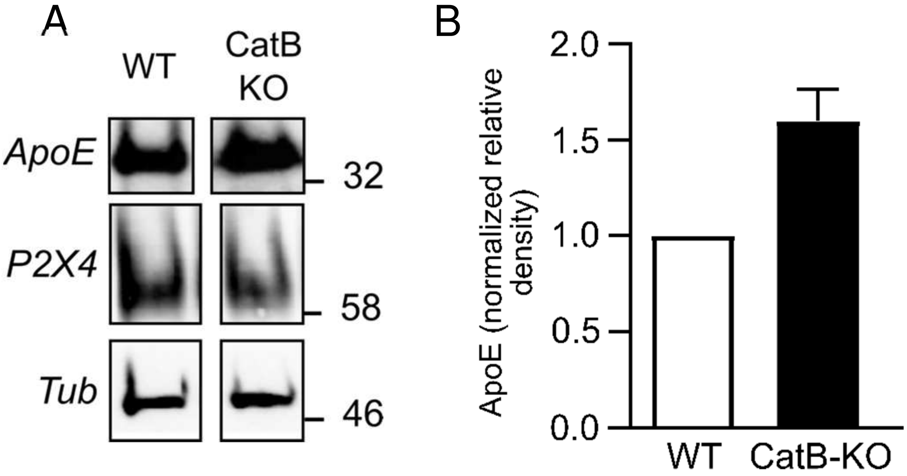
A
WT
CatB KO
ApoE
P2X4
Tub
32
58
46
B
ApoE (normalized relative density)
2.0
1.5
1.0
0.5
0.0
WT
CatB-KO

Sup Fig 9

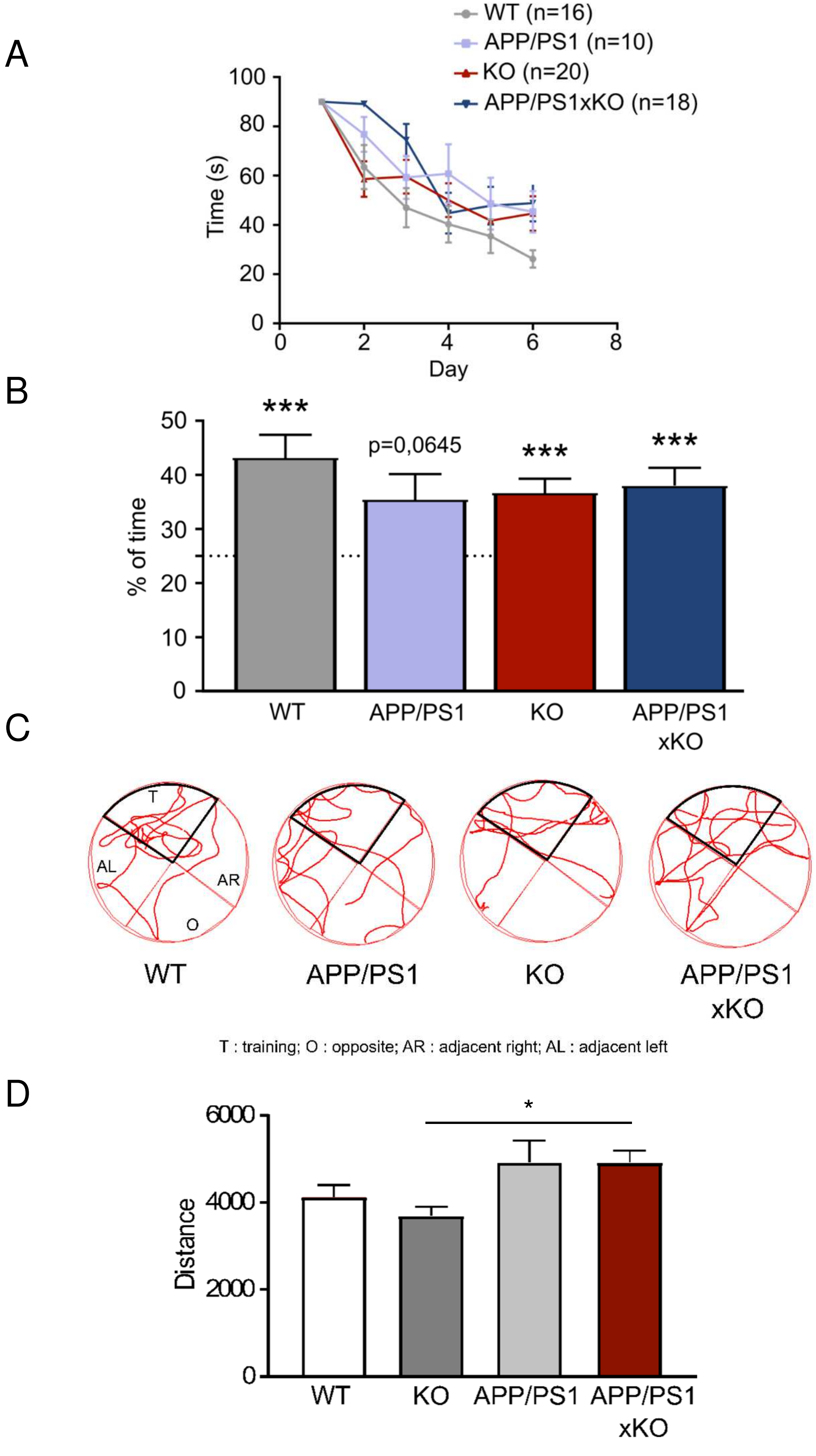
A
WT (n=16)
APP/PS1 (n=10)
KO (n=20)
APP/PS1xKO (n=18)
Time (s)
100
80
60
40
20
0
0
2
4
6
8
Day
B
***
p=0,0645
***
***
% of time
50
40
30
20
10
0
WT
APP/PS1
KO
APP/PS1 xKO
C
T
AL
AR
O
WT
APP/PS1
KO
APP/PS1 xKO
T : training; O : opposite; AR : adjacent right; AL : adjacent left
D
*
Distance
6000
4000
2000
0
WT
KO
APP/PS1
APP/PS1 xKO

Sup Fig 10

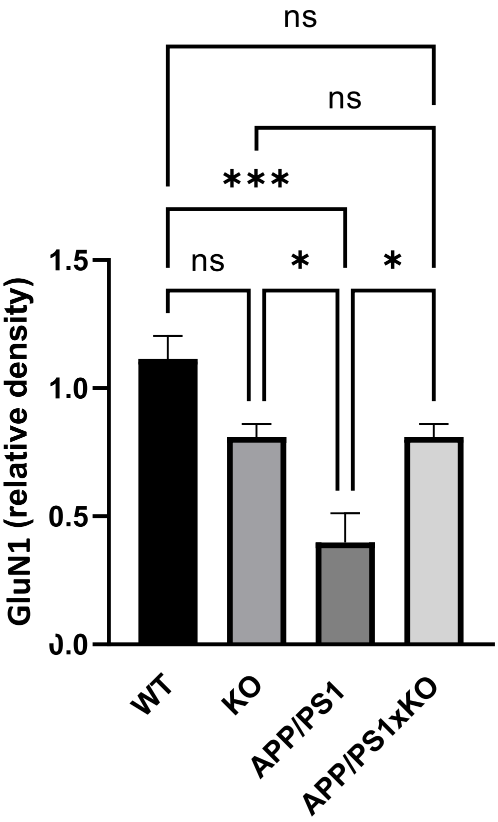
ns
ns
***
ns
*
*
GluN1 (relative density)
1.5
1.0
0.5
0.0
WT
KO
APP/PS1
APP/PS1xKO
